# Gut microbiota–derived metabolite trimethylamine-N-oxide and multiple health outcomes: an umbrella review and updated meta-analysis

**DOI:** 10.1093/ajcn/nqac074

**Published:** 2022-03-28

**Authors:** Doudou Li, Ying Lu, Shuai Yuan, Xiaxia Cai, Yuan He, Jie Chen, Qiong Wu, Di He, Aiping Fang, Yacong Bo, Peige Song, Debby Bogaert, Kostas Tsilidis, Susanna C Larsson, Huanling Yu, Huilian Zhu, Evropi Theodoratou, Yimin Zhu, Xue Li

**Affiliations:** Department of Big Data in Health Science, School of Public Health, Center of Clinical Big Data and Analytics of The Second Affiliated Hospital, Zhejiang University School of Medicine, Hangzhou, China; Department of Big Data in Health Science, School of Public Health, Center of Clinical Big Data and Analytics of The Second Affiliated Hospital, Zhejiang University School of Medicine, Hangzhou, China; Department of Big Data in Health Science, School of Public Health, Center of Clinical Big Data and Analytics of The Second Affiliated Hospital, Zhejiang University School of Medicine, Hangzhou, China; Unit of Cardiovascular and Nutritional Epidemiology, Institute of Environmental Medicine, Karolinska Institute, Stockholm, Sweden; Department of Nutrition and Food Hygiene, Beijing Key Laboratory of Environmental Toxicology, School of Public Health, Capital Medical University, Beijing, China; National Research Institute for Health and Family Planning, Beijing, China; Department of Big Data in Health Science, School of Public Health, Center of Clinical Big Data and Analytics of The Second Affiliated Hospital, Zhejiang University School of Medicine, Hangzhou, China; Department of Epidemiology & Biostatistics, School of Public Health, Zhejiang University School of Medicine, Hangzhou, China; Department of Epidemiology & Biostatistics, School of Public Health, Zhejiang University School of Medicine, Hangzhou, China; Department of Nutrition, School of Public Health, Sun Yat-sen University, Guangzhou, China; Jockey Club School of Public Health and Primary Care, The Chinese University of Hong Kong, Hong Kong, China; School of Public Health and Women's Hospital, Zhejiang University School of Medicine, Hangzhou, China; Centre for Inflammation Research, University of Edinburgh, Edinburgh, United Kingdom; Department of Epidemiology and Biostatistics, School of Public Health, Imperial College London, London, United Kingdom; Department of Hygiene and Epidemiology, University of Ioannina School of Medicine, Ioannina, Greece; Unit of Cardiovascular and Nutritional Epidemiology, Institute of Environmental Medicine, Karolinska Institute, Stockholm, Sweden; Unit of Medical Epidemiology, Department of Surgical Sciences, Uppsala University, Uppsala, Sweden; Department of Nutrition and Food Hygiene, Beijing Key Laboratory of Environmental Toxicology, School of Public Health, Capital Medical University, Beijing, China; Department of Nutrition, School of Public Health, Sun Yat-sen University, Guangzhou, China; Centre for Global Health, Usher Institute, University of Edinburgh, Edinburgh, United Kingdom; Cancer Research UK Edinburgh Centre, Medical Research Council Institute of Genetics and Cancer, University of Edinburgh, Edinburgh, United Kingdom; Department of Epidemiology & Biostatistics, School of Public Health, Zhejiang University School of Medicine, Hangzhou, China; Department of Big Data in Health Science, School of Public Health, Center of Clinical Big Data and Analytics of The Second Affiliated Hospital, Zhejiang University School of Medicine, Hangzhou, China

**Keywords:** umbrella review, updated meta-analyses, trimethylamine-N-oxide, TMAO, all-cause mortality, cardiovascular disease, hypertension, diabetes mellitus

## Abstract

**Background:**

Trimethylamine-N-oxide (TMAO) is a gut microbiota–derived metabolite produced from dietary nutrients. Many studies have discovered that circulating TMAO concentrations are linked to a wide range of health outcomes.

**Objectives:**

This study aimed to summarize health outcomes related to circulating TMAO concentrations.

**Methods:**

We searched the Embase, Medline, Web of Science, and Scopus databases from inception to 15 February, 2022 to identify and update meta-analyses examining the associations between TMAO and multiple health outcomes. For each health outcome, we estimated the summary effect size, 95% prediction CI, between-study heterogeneity, evidence of small-study effects, and evidence of excess-significance bias. These metrics were used to evaluate the evidence credibility of the identified associations.

**Results:**

This umbrella review identified 24 meta-analyses that investigated the association between circulating TMAO concentrations and health outcomes including all-cause mortality, cardiovascular diseases (CVDs), diabetes mellitus (DM), cancer, and renal function. We updated these meta-analyses by including a total of 82 individual studies on 18 unique health outcomes. Among them, 14 associations were nominally significant. After evidence credibility assessment, we found 6 (33%) associations (i.e., all-cause mortality, CVD mortality, major adverse cardiovascular events, hypertension, DM, and glomerular filtration rate) to present highly suggestive evidence.

**Conclusions:**

TMAO might be a novel biomarker related to human health conditions including all-cause mortality, hypertension, CVD, DM, cancer, and kidney function. Further studies are needed to investigate whether circulating TMAO concentrations could be an intervention target for chronic disease.

This review was registered at www.crd.york.ac.uk/prospero/ as CRD42021284730.

## Introduction

Trimethylamine-N-oxide (TMAO) is a gut microbiota metabolite derived from phosphatidylcholine, choline, betaine, and l-carnitine, which are abundant in seafoods, dairy products, egg yolks, muscle, and organ meats (1, 2). These nutrients can be hydrolyzed by trimethylamine (TMA) lyase from gut flora to form the TMAO precursor TMA, which is further oxidized by hepatic flavin monooxygenases to form TMAO (2, 3). A multitude of studies have discovered that circulating TMAO concentrations are linked to a wide range of health outcomes, including cardiovascular and cerebrovascular diseases (4–6), type 2 diabetes mellitus (DM) (7), hypertension (8), renal dysfunction (9, 10), cancer, and mortality (11, 12). The relations between elevated plasma TMAO concentrations and health-related traits have also been explored, including glomerular filtration rate (GFR) (9), blood pressure (13, 14), BMI (9, 14), and total cholesterol (15). It has been hypothesized that the intestinal microbiota may alter the risk of disease by inducing TMAO changes in the metabolome profile (16), and therefore TMAO might be a novel biomarker representing human health conditions related to the gut microbiota (17–19).

Most evidence on the health effects of plasma TMAO concentrations has been generated by observational studies with conflicting results. In addition, some studies were conducted among patients with specific diseases, which calls into question whether such associations can be generalized to a healthy population. Hence, it is necessary to synthesize the current evidence to provide a comprehensive overview of the claimed associations of TMAO concentrations with health outcomes.

Umbrella review is designed to provide a comprehensive overview of evidence from systematic review with or without meta-analysis (20). Several meta-analyses on the relations between increased TMAO concentrations and risks of obesity (21), stroke (22), diabetes (23), hypertension (24), and all-cause mortality (25) have been conducted. A comprehensive credibility assessment of these associations will help elucidate the role of TMAO in human health. Using a standardized approach, we performed an umbrella review to evaluate the validity and credibility of the evidence from updated meta-analyses of observational studies. In detail, we summarized the range of related health outcomes; presented the magnitude, direction, and significance of the reported associations; assessed the potential biases; and identified the most convincing evidence in relation to the health impact of TMAO concentrations.

## Methods

### Study design

In this umbrella review, all meta-analyses on the associations between plasma TMAO concentrations and health outcomes were identified. Original studies that evaluated the associations between TMAO and health outcomes were also identified to update the identified meta-analyses. The protocol of the present study was registered in PROSPERO (CRD42021284730).

### Literature search

Two investigators (DL and YL) independently searched the Embase, Medline, Web of Science, and Scopus databases from inception to 15 February, 2022 using a search strategy to identify meta-analyses of observational studies. The literature search algorithm was as follows: “((((meta-analysis) OR (meta)) OR (systematic overview)) OR (systematic review)) AND ((((trimethylamine oxide) OR (trimethylamine N-oxide)) OR (trimethylammonium oxide)) OR (TMAO)).” We also searched for individual observational studies to update the identified meta-analyses and reported the results in accordance with the Preferred Reporting Items for Systematic Reviews and Meta-Analyses (PRISMA) checklist (26). All identified publications went through a 3-step parallel review of title, abstract, and full text based on predefined inclusion and exclusion criteria, and any discrepancies were resolved by consensus.

### Eligibility criteria

Meta-analyses performing quantitative analysis of plasma TMAO concentrations and health outcomes were included in the umbrella review. All relevant population-based observational studies including prospective cohort, nested case–control, case cohort, case–control, or analytical cross-sectional studies were combined in the updated meta-analysis, and we conducted subgroup analysis by study design. Guidelines, narrative reviews, literature reviews, and genetic studies were excluded. We further excluded studies in which TMAO was not the primary exposure. Meta-analyses or original studies that had inadequate data (e.g., lack of information on RRs, ORs, HRs, or 95% CIs) were also excluded.

### Data extraction and quality assessment

From each eligible meta-analysis, we extracted information on the lead author's name, study design, publication year, study sample, number of studies included, the reported summary risk estimates [RR, OR, HR, or weighted mean difference (WMD)] with 95% CIs, the number of participants and cases, and the investigated outcomes. For meta-analyses on >1 health outcome, each outcome was recorded separately. Furthermore, we searched for recently published original articles on TMAO and combined them with studies identified from the previous meta-analyses to update the meta-analyses. When updating the meta-analyses, we added the newly identified studies and re-estimated the summary effect estimates using random-effects models. To account for potential confounding and reverse causality, we performed subgroup analyses by confining the meta-analyses to include only cohort studies with adjustment for renal function and diet (if possible). Data extraction at this stage covered information on study design, number of cases, total number of participants, RR estimates, and 95% CIs. Two investigators (DL and YL) extracted data independently using a predesigned data extraction form. The quality of individual studies was assessed by the Newcastle-Ottawa Scale (NOS) for observational studies (27).

### Statistical analysis

For each unique meta-analysis of observational studies, several metrics were estimated, including the summary effect and corresponding 95% CI using the random-effects model; the heterogeneity among studies (*Q* statistic and *I*^2^ metric); and the 95% prediction interval (95% PI) to predict the range of effect size that would be expected in a new original study after accounting for both the heterogeneity among individual studies and the uncertainty of the summary effect estimated in the random-effects model (28) (the calculation of the 95% PI is based on the predicted distribution derived from a function of the degree of heterogeneity, number of studies included, and within-study SEs) (29, 30). Egger's regression test was used to evaluate the small-study effects (31). The excess significance test was conducted to investigate whether the observed number of studies with significant results differed from the expected number of significant studies using the *χ*^2^ test (32–34). The expected number of significant studies for each meta-analysis was calculated by summing the statistical power estimates for each component study. We estimated the power of each study for an effect equal to the effect of the largest study (the study with the smallest variance), as previously described (35). All statistical analyses were performed using the “metafor” and “forestplot” R packages, R software version 4.0.2 (The R Foundation, Boston, MA).

### Evaluation of evidence credibility

We used credibility assessment criteria (**Supplemental Table 1**), as described in previously published umbrella reviews (35–37). Evidence from meta-analyses of observational studies with nominally significant summary results (*P* < 0.05) was classified into 4 categories: convincing, highly suggestive, suggestive, or weak evidence (class I, II, III, and IV, respectively) (35–37). For meta-analyses performed on the same outcome, we examined the consistency between studies and the largest meta-analysis was retained for further analyses.

## Results

**FIGURE 1:**
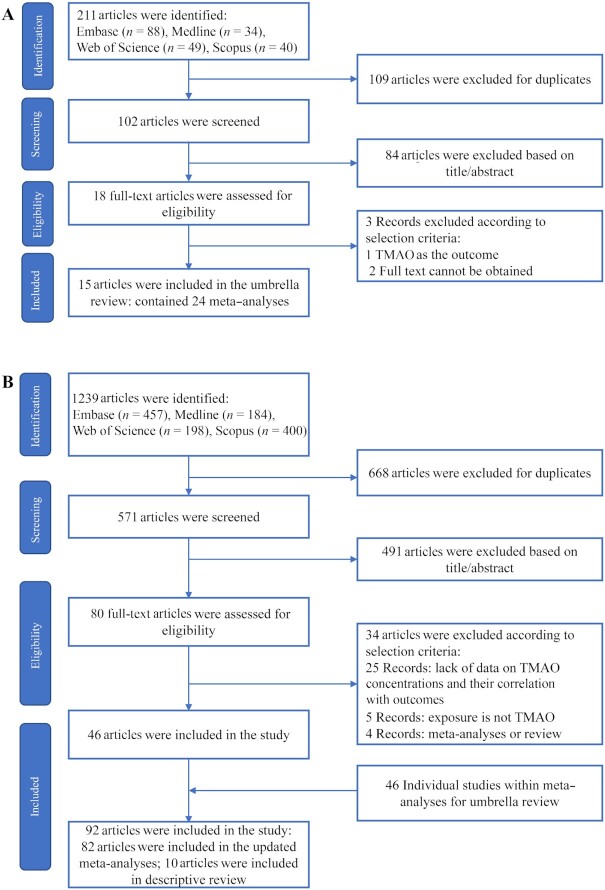
Flow diagram of study selection. (A) Study selection for umbrella review; (B) study selection for the updated meta-analyses. TMAO, trimethylamine N-oxide.

**Figure 1**A shows the process of literature searching and screening for the umbrella review. The literature search retrieved 211 unique articles. After literature screening, 15 articles (21–25, 38–47) were eligible, which contained 24 meta-analyses for 15 unique outcomes (**Supplemental Table 2**). There was 1 meta-analysis published for stroke (22), hypertension (42), diastolic blood pressure (DBP) (24), systolic blood pressure (SBP) (24), diabetes (23), BMI (21), LDL/HDL cholesterol (24), total cholesterol (TC) (24), triglycerides (24), C-reactive protein (CRP) (41), and GFR (47); there were 2 meta-analyses for cardiovascular disease (CVD) (39, 46); 5 meta-analyses for all-cause mortality (25, 38–40, 45); and 6 meta-analyses for major adverse cardiovascular events (MACE) (25, 38, 43–45).

Figure 1B shows the process of selection of original studies in conducting the updated meta-analyses. The initial search yielded 1239 publications. After literature screening, we retrieved 46 new articles; and together with 46 individual studies from previous meta-analyses, a total of 92 individual studies were included in the study. Among them, 82 individual studies were included in the meta-analyses. The updated meta-analyses evaluated the associations between plasma TMAO concentrations and 18 unique health outcomes. **Supplemental Tables 3–5** show the quality assessment of the included studies.

### All-cause mortality

**FIGURE 2:**
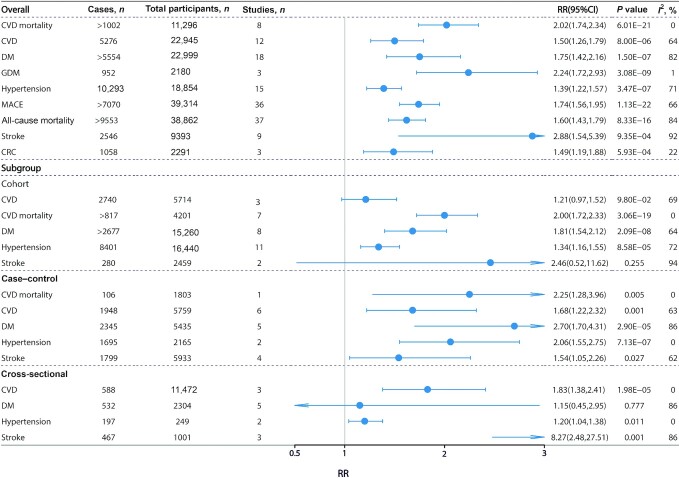
High compared with low TMAO concentrations and associations with multiple health outcomes. Estimates are RRs and meta-analyses are based on random-effect models. An *I*^2^ value ≥50% is considered to indicate substantial heterogeneity. All results are presented as HR with 95% CIs, using the Mantel–Haenszel method with a random-effects model. CRC, colorectal cancer; CVD, cardiovascular disease; DM, diabetes mellitus; GDM, gestational diabetes mellitus; MACE, major adverse cardiovascular events.

**FIGURE 3:**
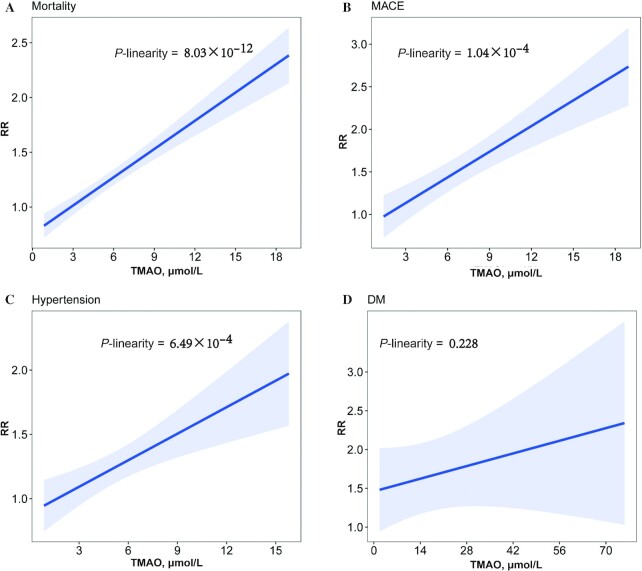
Dose–response association between circulating TMAO concentrations and all-cause mortality (A), MACE (B), hypertension (C), and DM (D). Risk spline (solid line) and 95% CIs (shadow) of pooled RR of all-cause mortality, MACE, hypertension, and DM by 1 μmol/L of TMAO. DM, diabetes mellitus; MACE, major adverse cardiovascular events; TMAO, trimethylamine N-oxide.

The updated meta-analysis included 37 studies from 32 articles (3, 5, 10–12, 48–74) with >9553 cases out of 38,862 participants. All-cause mortality in the highest TMAO category was compared with that in the lowest TMAO category, and it was found that a higher TMAO concentration was associated with higher mortality (HR: 1.60; 95% CI: 1.43, 1.79; *P* = 8.33 × 10^−16^) (**Figure 2**, **Supplemental Figure 1**). A dose-response meta-analysis based on 10 studies (3, 5, 10, 12, 58, 62, 65, 66, 68, 70) showed that a 1-unit increment of TMAO (1 μmol/L) was associated with a 9% increased risk of all-cause mortality (HR: 1.09; 95% CI: 1.07, 1.11; *P* = 8.03 × 10^−12^) (**Figure 3**A). We also conducted a subgroup analysis by disease status and found that the association between TMAO and all-cause mortality was predominant in CVD patients (HR: 1.66; 95% CI: 1.46, 1.88; *P* = 1.84 × 10^−15^) (**Supplemental Figure 2**), whereas no significant association was reported in other populations. The association with all-cause mortality remained significant when including only the studies that adjusted for renal function (HR: 1.56; 95% CI: 1.38, 1.77; *P* = 3.45 × 10^−12^) (**Supplemental Figure 3**).

### Cardiovascular outcomes

Regarding MACE, 36 studies from 32 articles (2, 5, 10, 48–53, 55–61, 63, 65, 66, 68, 70, 75–85) were included in the updated meta-analysis, contributing >7070 cases in 39,314 participants. In the random-effects model, circulating TMAO was positively associated with an increased risk of MACE (HR: 1.74; 95% CI: 1.56, 1.95; *P* = 1.13 × 10^−22^) (Figure 2, **Supplemental Figure 4**). The association remained significant in the confined meta-analysis of cohort studies that adjusted for renal function (HR: 1.65; 95% CI: 1.45, 1.88; *P* = 1.50 × 10^−14^) (**Supplemental Figure 5**). Three studies (66, 68, 70) were included in the dose-response analysis, resulting in 11% increased risk of MACE per 1-μmol/L increment of TMAO (RR: 1.11; 95% CI: 1.07, 1.14; *P* = 1.04 × 10^−4^) (Figure 3B).

Fifteen studies (3, 15, 53, 55, 58, 65, 66, 77, 83, 84, 86–90) were included in the updated meta-analysis of hypertension, comprising 10,293 cases and 18,854 total participants. There was a significant association between TMAO concentrations and risk of hypertension (RR: 1.39; 95% CI: 1.22, 1.57; *P* = 3.47 × 10^−7^) (Figure 2, **Supplemental Figure 6**), which was consistent with a former published meta-analysis (42). The association remained significant in the confined meta-analysis of cohort studies only (RR: 1.34; 95% CI: 1.16, 1.55; *P* = 8.58 × 10^−5^) (Figure 2), and the association was still significant when the meta-analysis included only the studies that adjusted for renal function (RR: 1.40; 95% CI: 1.13, 1.72; *P* = 1.65 × 10^−3^) (**Supplemental Figure 7**). Eight studies (3, 53, 55, 58, 66, 87–89) were eligible for dose-response analysis, which showed that the risk of hypertension increased by 7% per (1-μmol/L) increment of TMAO (RR: 1.07; 95% CI: 1.03, 1.11; *P* = 6.49 × 10^−4^) (Figure 3C).

The updated meta-analysis on CVDs included 12 studies (4, 6, 83, 91–96) with 22,945 participants and showed that high TMAO concentrations were statistically significantly associated with an increased risk of CVD (OR: 1.50; 95% CI: 1.26, 1.79; *P* = 8.00 × 10^−6^) (Figure 2, **Supplemental Figure 8**). Eight studies from 5 articles (11, 14, 60, 72, 83) were used to perform a meta-analysis on CVD mortality. The results revealed that participants with high TMAO concentrations were more likely to die from CVDs than those with low TMAO concentrations (HR: 2.02; 95% CI: 1.74, 2.34; *P* = 6.01 × 10^−21^) (Figure 2, **Supplemental Figure 9**). The association remained significant when the meta-analysis was restricted to cohort studies (HR: 2.00; 95% CI: 1.72, 2.33; *P* = 3.06 × 10^−19^) (Figure 2).

Results from the updated meta-analysis of stroke showed that higher circulating TMAO concentrations were associated with a higher risk of stroke [9 studies (66, 69, 83, 90, 97–100) enrolling 9393 participants, OR: 2.88; 95% CI: 1.54, 5.39; *P* = 9.35 × 10^−4^] (Figure 2, **Supplemental Figure 10**). However, this association was attenuated and not significant when the meta-analysis was restricted to cohort studies (RR: 2.46; 95% CI: 0.52, 11.62; *P =* 0.255) (Figure 2).

### DM

Our updated meta-analyses, including 18 studies [from 17 articles (3, 7, 15, 55, 65, 77, 83, 84, 86, 87, 90, 93, 101–105) enrolling 22,999 subjects], found a significant association between TMAO and DM (OR: 1.75; 95% CI: 1.42, 2.16; *P* = 1.50 × 10^−7^) (Figure 2, **Supplemental Figure 11**). The association was also significant in the confined meta-analysis of cohort studies (OR: 1.81; 95% CI: 1.54, 2.12; *P* = 2.09 × 10^−8^) (Figure 2), and the association remained significant when the meta-analysis was restricted to cohort studies that adjusted for renal function (OR: 1.71; 95% CI: 1.35, 2.18; *P* = 1.12 × 10^−5^) (**Supplemental Figure 12**). In our dose-response meta-analysis, based on data from 3 articles (87, 88, 102), we found no statistically significant relation between TMAO concentrations and DM (*P* = 0.228) (Figure 3D). Furthermore, our meta-analysis of 3 studies enrolling 2180 subjects showed that women with high TMAO concentrations were more likely to have gestational diabetes mellitus (GDM) (OR: 2.24; 95% CI: 1.72, 2.93; *P* = 3.08 × 10^−9^) (Figure 2, **Supplemental Figure 13**).

### Cancer risk

We identified 6 observational studies that examined the associations of TMAO concentrations with cancer risk including colorectal cancer (CRC) (106–108), prostate cancer (109), primary liver cancer (110), and pancreatic cancer (111). Quantitative meta-analysis could only be performed for CRC, which included 3 individual studies and showed a positive association between high TMAO concentrations and increased risk of CRC (OR: 1.49; 95% CI: 1.19, 1.88; *P* = 5.93 × 10^−4^) (Figure 2, **Supplemental Figure 14**). Three articles reported positive associations of TMAO with prostate cancer (OR: 1.36; 95% CI: 1.02, 1.81; *P* = 0.039) (109), primary liver cancer (OR: 2.85; 95% CI: 1.59, 5.11; *P* = 0.003) (110), and pancreatic cancer (OR: 2.36; 95% CI: 1.30, 4.26; *P* = 0.02) (111) (**Supplemental Table 6**).

### Blood pressure and cardiometabolic biomarkers

**FIGURE 4:**
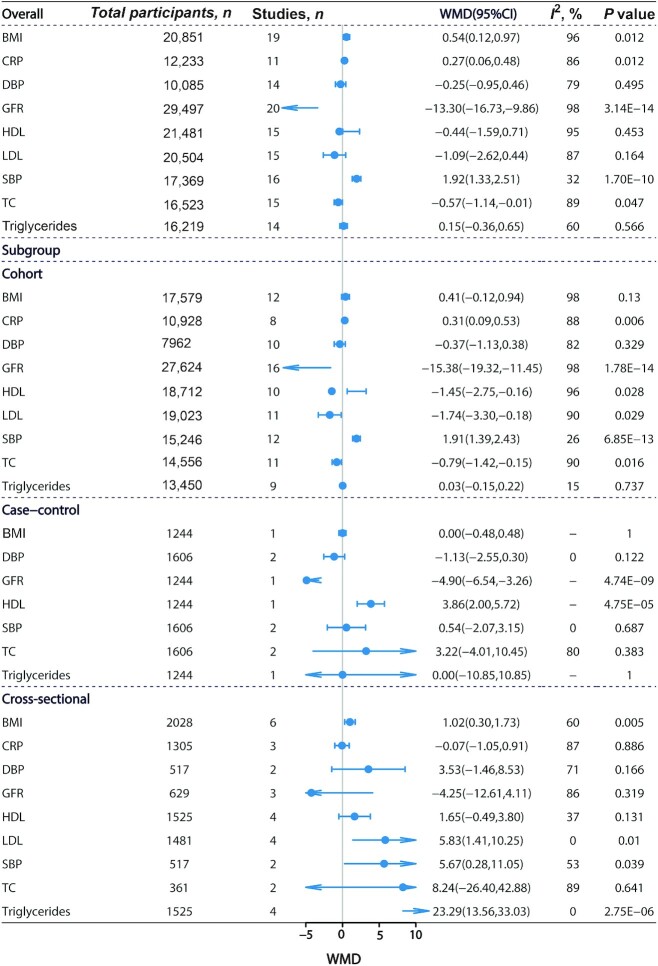
High compared with low TMAO concentrations and associations with multiple health outcomes. Estimates are WMD and meta-analyses are based on random-effect models. An *I*^2^ value ≥50% is considered to indicate substantial heterogeneity. All results are presented as HR with 95% CIs, using the Mantel–Haenszel method with a random-effects model. CRP, triglycerides and C-reactive protein; DBP, diastolic blood pressure; GFR, glomerular filtration rate; HDL, HDL cholesterol; LDL, LDL cholesterol; SBP, systolic blood pressure; TC, total cholesterol; WMD, weighted mean difference.

The results of the updated meta-analyses showed no significant association between TMAO and DBP [14 studies (9, 13–15, 59, 67, 87–89, 98, 112–114) enrolling 10,085 subjects, WMD: −0.25; 95% CI: −0.95, 0.46; *P* = 0.495] (**Figure 4**, **Supplemental Figure 15**). Higher circulating TMAO was related to higher SBP [16 studies (3, 9, 13–15, 59, 67, 87–89, 98, 112–115) enrolling 17,369 subjects, WMD: 1.92; 95% CI: 1.33, 2.51; *P* = 1.70 × 10^−10^] (Figure 4, **Supplemental Figure 16**) and BMI [19 studies (3, 9, 13, 14, 53, 65, 67, 84, 87–90, 98, 103, 113–116) enrolling 20,851 subjects, WMD: 0.54; 95% CI: 0.12, 0.97; *P* = 0.012] (Figure 4, **Supplemental Figure 17**). The association between TMAO concentrations and SBP remained significant when the meta-analysis included only cohort studies (WMD: 1.91; 95% CI: 1.39, 2.43; *P* = 6.85 × 10^−13^) (Figure 4).

The updated meta-analyses showed that high TMAO concentrations were associated with increased CRP concentrations (WMD: 0.27; 95% CI: 0.06, 0.48; *P* = 0.012) (Figure 4, **Supplemental Figure 18**) and decreased concentrations of TC (WMD: −0.57; 95% CI: −1.14, −0.01; *P* = 0.047) (Figure 4, **Supplemental Figure 19**) but not of other lipids (HDL cholesterol, LDL cholesterol, triglycerides) (Figure 4, **Supplemental Figures 20–22**). The associations between TMAO concentrations and CRP (WMD: 0.31; 95% CI: 0.09, 0.53; *P* = 0.006) (Figure 4), HDL cholesterol (WMD: −1.45; 95% CI: −2.75, −0.16; *P* = 0.028) (Figure 4), LDL cholesterol (WMD: −1.74; 95% CI: −3.30, −0.18; *P* = 0.029) (Figure 4), and TC (WMD: −0.79; 95% CI: −1.42, −0.15; *P* = 0.016) (Figure 4) were significant in the confined meta-analyses of cohort studies.

### Renal function

The umbrella review identified 1 meta-analysis reporting that circulating TMAO was associated with a decrease of GFR (WMD: −12.86; 95% CI: −16.57, −9.15; *P* = 1.11 × 10^−11^) (47). Our updated meta-analysis including 20 studies from 19 articles (3, 9, 13, 14, 48, 53, 55, 59, 65–67, 70, 77, 80, 89, 98, 113–115) enrolling 29,497 subjects found a consistently significant association (WMD: −13.30; 95% CI: −16.73, −9.86; *P* = 3.14 × 10^−14^) (Figure 4, **Supplemental Figure 23**). The association remained significant in the confined meta-analysis of cohort studies (WMD: −15.38; 95% CI: −19.32, −11.45; *P* = 1.78 × 10^−14^) (Figure 4).

### Other health outcomes

We identified 10 original articles (17, 109–111, 117–122) that reported associations between TMAO concentrations and other health outcomes (Figure 1B, Supplemental Table 6). One reported that TMAO was not significantly associated with the risk of pre-eclampsia (117). Others reported significant associations between TMAO concentrations and other health outcomes [metabolic syndrome (17), diabetic retinopathy (118), hip fracture (119), Parkinson disease (120), and nonalcoholic fatty liver disease (121, 122)]. Quantitative meta-analysis could not be performed owing to the limited number of studies identified for these health outcomes.

### Evidence assessment of included studies

Evidence assessment of the identified associations was performed according to our credibility assessment criteria (Supplemental Table 1, **Table 1**). Eight (44%) meta-analyses had *P* < 10^−6^, 6 (33%) had a 95% PI that excluded the null, 12 (67%) had >1000 cases (or >20,000 total participants for continuous outcomes), 5 (28%) had no large heterogeneity (*I*^2^ < 50%), and 11 (61%) had neither small-study effects nor excess significance bias. After credibility assessment, no outcome presented convincing evidence; 6 (33%) health outcomes presented highly suggestive evidence (class II: CVD mortality, hypertension, MACE, all-cause mortality, DM, GFR); 3 (17%) presented suggestive evidence (class III: stroke, CVD, and CRC); and 5 (28%) presented weak evidence (class IV: SBP, BMI, TC, CRP, and GDM) for their associations with circulating TMAO concentrations.

**TABLE 1:** Association between TMAO concentrations and health outcomes and evidence class for meta-analyses^1^

Outcomes	Population	Study design	Comparison	Studies, *n*	Cases, *n*	Participants, *n*	Metric	Random-effects RR/HR/OR/WMD (95% CI)	*P* value	95% PI	Egger's *P*^2^	*I* ^2^,^3^ %	*P* value for excess significance test^4^	Evidence class^5^
Cardiovascular outcomes														
CVD	CVD/non-CVD	CC/CS	High vs. low	12	5276	22,945	OR	1.50 (1.26, 1.79)	8.00E−06	0.92, 2.44	0.000	63.97	0.16	III
Hypertension	Healthy/hypertension	CO/CC/CS	High vs. low	15	10,293	18,854	RR	1.39 (1.22, 1.57)	3.47E−07	0.97, 1.99	0.201	70.99	NP	II
MACE	CKD/CVD/DM	CO/CC/CS	High vs. low	36	>7070	39,314	HR	1.74 (1.55, 1.95)	1.13E−22	1.07, 2.82	0.011	65.59	0.00	II
Stroke	Stroke/CVD/DM	CO/CC/CS	High vs. low	9	2546	9393	OR	2.88 (1.54, 5.39)	9.35E−04	0.44, 18.81	0.439	91.54	NP	III
Mortality														
All-cause mortality	General/CVD/CKD/DM	CO	High vs. low	37	>10,510	44,480	HR	1.60 (1.43, 1.79)	8.33E−16	0.91, 2.82	0.000	83.63	0.10	II
CVD mortality	CVD/non-CVD	CO/CC	High vs. low	8	>1002	11,296	HR	2.02 (1.74, 2.34)	6.01E−21	1.74, 2.34	0.480	0.00	0.24	II
Blood pressure and cardiometabolic biomarkers														
SBP	General/DM/CVD/stroke	CO/CC/CS	High vs. low	16	NA	17,369	WMD	1.92 (1.33, 2.51)	1.70E−10	0.74, 3.10	0.530	32.42	0.45	IV
DBP	General/DM/CVD/stroke	CO/CC/CS	High vs. low	14	NA	10,085	WMD	−0.25 (−0.95, 0.46)	0.495	−2.07, 1.57	0.338	79.15	0.85	NS
BMI	General/DM/CVD/stroke	CO/CC/CS	High vs. low	19	NA	20,851	WMD	0.54 (0.12, 0.97)	0.012	−1.11, 2.20	0.954	96.13	0.18	IV
HDL cholesterol	General/DM/CVD/stroke	CO/CC/CS	High vs. low	15	NA	21,481	WMD	−0.44 (−1.59, 0.71)	0.453	−4.42, 3.54	0.151	94.91	NP	NS
LDL cholesterol	General/DM/CVD/stroke	CO/CC/CS	High vs. low	15	NA	20,504	WMD	−1.09 (−2.62, 0.44)	0.164	−5.31, 3.14	0.286	87.44	0.70	NS
TC	General/DM/CVD/stroke	CO/CC/CS	High vs. low	15	NA	16,523	WMD	−0.57 (−1.14, −0.01)	0.047	−2.16, 1.02	0.513	88.65	0.65	IV
CRP	General/DM/CVD/stroke	CO/CS	High vs. low	11	NA	12,233	WMD	0.27 (0.06, 0.48)	0.012	−0.27, 0.81	0.112	86.11	NP	IV
Triglycerides	General/DM/CVD	CO/CC/CS	High vs. low	14	NA	16,219	WMD	0.15 (−0.36, 0.65)	0.566	−0.83, 1.13	0.000	60.02	0.76	NS
Diabetes mellitus														
Diabetes	CVD/diabetes/renal disease	CO/CC/CS	High vs. low	18	>5554	22,999	OR	1.75 (1.42, 2.16)	1.50E−07	0.83, 3.70	0.886	82.05	0.10	II
GDM	GDM/non-GDM	CC	High vs. low	3	952	2180	OR	2.24 (1.72, 2.93)	3.08E−09	1.71, 2.94	0.240	0.94	0.78	IV
Renal outcomes														
GFR	CKD/general	CO/CC/CS	High vs. low	20	NA	29,497	WMD	−13.30 (−16.73, −9.86)	3.14E−14	−28.65, 2.05	0.724	97.92	0.76	II
Cancer														
CRC	CRC/non-CRC	CC	High vs. low	3	1058	2291	OR	1.49 (1.19, 1.88)	5.93E−04	1.11, 2.00	0.194	21.72	0.65	III

1 CC, case–control study; CKD, chronic kidney disease; CO, cohort study; CRC, colorectal cancer; CRP, C-reactive protein; CS, cross-sectional study; CVD, cardiovascular disease; DBP, diastolic blood pressure; DM, diabetes mellitus; GDM, gestational diabetes mellitus; GFR, glomerular filtration rate; MACE, major adverse cardiovascular events; NA, not available; NP, not pertinent (because the number of expected significant studies was larger than the number of observed significant studies); NS, not significant; PI, prediction interval; SBP, systolic blood pressure; TC, total cholesterol; WMD, weighted mean difference.

2 Egger's regression test was used to evaluate the small-study effects.

3 Interstudy heterogeneity was tested using the Cochran *Q* statistic (*t*^2^) at a significance level of *P* < 0.10 and quantified by the *I^2^* statistic. An *I*^2^ value ≥50% is considered to indicate substantial heterogeneity. All results are presented as RR/OR/HR/WMD with 95% CIs, using the Mantel–Haenszel method with a random-effects model.

4 Excess significance test was conducted to investigate whether the observed number of studies with significant results differed from the expected number of significant studies using the χ^2^ test.

5 Evidence class criteria: class I (convincing): statistical significance with *P* < 10^−6^, >1000 cases (or >20,000 participants for continuous outcomes), the largest component study reported a statistically significant effect (*P* < 0.05), 95% PI excluded the null, no large heterogeneity (*I*^2^ < 50%), no evidence of small-study effects (*P* > 0.10) or excess significance bias (*P* > 0.10); class II (highly suggestive): statistical significance with *P* < 10^−6^, >1000 cases (or >20,000 participants for continuous outcomes), the largest component study reported a statistically significant effect (*P* < 0.05); class III (suggestive): statistical significance with *P* < 10^−3^, >1000 cases (or >20,000 participants for continuous outcomes); class IV (weak): the remaining statistically significant associations with *P* < 0.05.

## Discussion

Our updated meta-analyses included a total of 82 individual studies and examined the associations of TMAO with 18 unique health outcomes. Among them, 14 outcomes (all-cause mortality, CVD, MACE, stroke, hypertension, CVD mortality, SBP, BMI, CRP, TC, DM, GDM, GFR, CRC) were found to be significantly associated with TMAO concentrations. When we restricted meta-analyses to only include cohort studies, 11 outcomes (all-cause mortality, MACE, hypertension, CVD mortality, SBP, CRP, HDL cholesterol, LDL cholesterol, TC, DM, GFR) were still significantly associated with TMAO concentrations. The dose-response analyses revealed that circulating TMAO concentrations were positively associated with the risk of hypertension and MACE. After assessment of the evidence credibility, we found highly suggestive associations of TMAO concentrations with 6 health outcomes, including all-cause mortality, CVD mortality, MACE, hypertension, DM, and GFR.

Former published meta-analyses (25, 38–40, 45) demonstrated that high TMAO concentrations were related to an increased risk of all-cause mortality and the updated meta-analysis showed consistent results. When conducting subgroup analysis by disease status, TMAO showed a significant association with all-cause mortality only in patients with CVD. In addition, our study revealed a positive association between TMAO concentrations and CVD risk. Given that the majority of evidence was from case–control studies, we cannot rule out reverse causality. It has been reported that TMAO may affect platelet reactivity, lipid metabolism, and endothelial dysfunction, which could result in the acceleration of atherosclerotic plaque formation (123). Because atherosclerosis is one of the major causes of CVD, high concentrations of TMAO could be related to high incidence of CVD, due to TMAO's contribution in the development of atherosclerosis. However, no causal association between TMAO and CVD was identified in a recent bidirectional Mendelian randomization study (124). Taken together, current evidence suggests that TMAO might be a novel biomarker indicating the risk of CVD.

Our umbrella review reported a highly suggestive association between TMAO concentrations and hypertension, and both the former published study (42) and the updated meta-analysis revealed that this association displayed a dose–response relation. Previous studies have found that hypertensive patients had more gut microbial enzymes involved in TMA production than those without hypertension (125). Animal studies have also found that elevated plasma concentrations of TMAO can prolong the duration of elevated blood pressure (126–128). TMAO could also promote Ang II–induced vasoconstriction via the PERK/ROS/CaMKII/PLCβ3 (protein kinase r-like endoplasmic reticulum kinase (PERK), reactive oxygen species (ROS), calmodulin-dependent protein kinase ΙΙ (CaMKΙΙ), phospholipase c β3 (PLCβ3) axis, thereby facilitating Ang II–induced hypertension (126).

Both the former published study (23) and the updated meta-analysis revealed a positive association between TMAO concentrations and risk of DM. Previous studies reported supportive evidence on associations between TMAO and diabetes-related traits, including insulin resistance, impaired glucose metabolism, and metabolic syndrome (17, 129, 130). Animal studies also found that TMAO may exacerbate impaired glucose tolerance and hyperglycemia by blocking the hepatic insulin signaling pathway and causing inflammation in adipose tissue (131), whereas a decrease of plasma TMAO could reduce plasma glucose and insulin resistance in mice by inhibiting the main TMAO-generating enzyme FMO3 (flavin-containing monooxygenase-3) (132). Furthermore, we found evidence from 2 studies (133, 134) reporting a positive association between TMAO concentrations and GDM, but the involvement of TMAO in any causal or compensatory pathway has not been proven. Therefore, further studies should be conducted to understand the mechanism of TMAO influencing GDM.

The former published study (47) and updated meta-analysis showed that an increase of TMAO concentrations was associated with lower GFR. Previous studies showed that chronic dietary exposures that increased TMAO concentrations appeared to directly contribute to progressive renal fibrosis and dysfunction (10, 135), which is one of the main end-stage renal diseases and a common outcome of almost all progressive chronic kidney diseases (CKDs) (136). Animal studies demonstrated that inhibition of TMAO production attenuated CKD development and cardiac hypertrophy in mice, suggesting that TMAO concentrations may play an important role in CKD development and TMAO reduction may be a novel strategy in treating CKD and its CVD complications (137). However, in this umbrella review, we only assessed the observational association of TMAO with GFR as an intermediate surrogate trait of CKD. Future studies focusing on CKD as an endpoint need to be performed to examine the association with TMAO concentrations.

It is widely known that TMAO is produced from the fermentation of dietary nutrients (choline, betaine, and carnitine) by the gut microbiota. Considering high concentrations of TMAO being associated with gut microbiota balance and several diseases, nonpharmacologic strategies, including foods and dietary supplements rich in bioactive compounds or nutrients, have the potential to modulate the gut microbiota to reduce TMAO concentrations, and therefore decrease the risk of several diseases. There is evidence showing that TMAO concentrations can be reduced by some bioactive compounds, such as resveratrol, allicin, capsanthin, and dietary components present in the apple, oolong tea, natural wheat bran, and low-fat diet, whereas strategies such as the paleolithic diet, high-fat diet, and high-protein diet promote increased TMAO concentrations (138). Because TMAO is a metabolite produced by the gut microbiota, targeting the gut microbiota and the metabolic pathway of TMAO might provide new strategies for the prevention of these related diseases (139). Further studies should be conducted to evaluate these dietary components’ effectiveness, dose, and intervention time on TMAO concentrations and whether their health effects could be mediated through regulating TMAO concentrations.

### Study strengths and limitations

Although previous meta-analyses of TMAO and the risk of disease outcomes have been conducted, our study is the first to summarize and present the evidence for the associations between TMAO concentrations and a wide spectrum of health outcomes systematically and thoroughly by incorporating information from meta-analyses of observational studies. In addition, our dose-response analyses revealed that there were no critical concentrations of TMAO in terms of varying degrees of risk in patients with all-cause mortality, diabetes, hypertension, and MACE disease. Subgroup analyses further evaluated the associations by only including prospective studies or studies adjusted for certain confounding factors. Although previous studies reported multiple health outcomes associated with TMAO concentrations, our study evaluated the reliability of these associations based on established credibility criteria.

Our study also has limitations. First, because all the included studies were observational, causal associations between circulating TMAO and related outcomes cannot be inferred. Second, sex- and ethnicity-specific findings could not be obtained owing to limited data. Diet-specific findings could not be obtained owing to limited data, and therefore we were not able to perform subgroup analyses to further explore the associations by minimizing the potential confounding of dietary patterns. Third, there was high heterogeneity in the current meta-analyses, possible reasons being the inclusion of different populations and different study designs. Further, our evidence grading was not sensitive to the use of 95% PIs or excess significance bias because the evidence grading remained the same when we removed them consecutively. In addition, when updating the meta-analyses, we added the newly identified studies, re-estimated summary effect estimates using random-effects models, and applied a set of well-established criteria to properly classify the evidence according to the reported *P* values, heterogeneity, and excess significance bias, with consideration of the inflated risk of false positives inherited by the updated meta-analyses (140). Finally, the underlying mechanisms between TMAO and the development of various diseases have not been explored in depth.

### Conclusions

In conclusion, our umbrella review and updated meta-analyses identified multiple health outcomes associated with TMAO concentrations. Evidence assessment demonstrated that TMAO concentrations are associated with several health conditions, including all-cause mortality, CVD, hypertension, diabetes, and CKD. Our dose-response meta-analyses indicated that there were no critical concentrations of TMAO in terms of its health impact. Further studies are needed to investigate whether circulating TMAO concentrations could be an intervention target for chronic disease.

## Supplementary Material

nqac074_Supplemental_File

## Data Availability

All data relevant to the study are included in the article or as supplementary information.

## Notes

XL was supported by Natural Science Fund for Distinguished Young Scholars of Zhejiang Province grant LR22H260001. ET was supported by Cancer Research UK Career Development Fellowship C31250/A22804. SCL was supported by Swedish Heart Lung Foundation (Hjärt-Lungfonden) grant 20210351, Swedish Research Council (Vetenskapsrådet) grant 2019-00977, and the Swedish Cancer Society (Cancerfonden).

Supplemental Tables 1–6 and Supplemental Figures 1–23 are available from the “Supplementary data” link in the online posting of the article and from the same link in the online table of contents at https://academic.oup.com/ajcn/.

DL, YL, and SY contributed equally to this work as first authors.

ET, YZ, and XL contributed equally to this work as last authors.

Abbreviations used: CKD, chronic kidney diseases; CRC, colorectal cancer; CRP, C-reactive protein; CVD, cardiovascular disease; DBP, diastolic blood pressure; DM, diabetes mellitus; GDM, gestational diabetes mellitus; GFR, glomerular filtration rate; MACE, major adverse cardiovascular events; PI, prediction interval; SBP, systolic blood pressure; TC, total cholesterol; TMA, trimethylamine; TMAO, trimethylamine-N-oxide; WMD, weighted mean difference.

## References

[1] Hazen SL , Brown JM. Eggs as a dietary source for gut microbial production of trimethylamine-*N*-oxide. Am J Clin Nutr. 2014;100(3):741–3. 25080455 10.3945/ajcn.114.094458 PMC4135484

[2] Koeth RA , Wang Z, Levison BS, Buffa JA, Org E, Sheehy BT et al. Intestinal microbiota metabolism of l-carnitine, a nutrient in red meat, promotes atherosclerosis. Nat Med. 2013;19(5):576–85. 23563705 10.1038/nm.3145 PMC3650111

[3] Gruppen EG , Garcia E, Connelly MA, Jeyarajah EJ, Otvos JD, Bakker SJL et al. TMAO is associated with mortality: impact of modestly impaired renal function. Sci Rep. 2017;7(1):13781. 29061990 10.1038/s41598-017-13739-9 PMC5653802

[4] Heianza Y , Ma W, DiDonato JA, Sun Q, Rimm EB, Hu FB et al. Long-term changes in gut microbial metabolite trimethylamine N-oxide and coronary heart disease risk. J Am Coll Cardiol. 2020;75(7):763–72. 32081286 10.1016/j.jacc.2019.11.060 PMC8140616

[5] Li XS , Obeid S, Klingenberg R, Gencer B, Mach F, Räber L et al. Gut microbiota-dependent trimethylamine N-oxide in acute coronary syndromes: a prognostic marker for incident cardiovascular events beyond traditional risk factors. Eur Heart J. 2017;38(11):814–24. 28077467 10.1093/eurheartj/ehw582 PMC5837488

[6] Mei Z , Chen G-C, Wang Z, Usyk M, Yu B, Baeza YV et al. Dietary factors, gut microbiota, and serum trimethylamine-*N*-oxide associated with cardiovascular disease in the Hispanic Community Health Study/Study of Latinos. Am J Clin Nutr. 2021;113(6):1503–14. 33709132 10.1093/ajcn/nqab001 PMC8168354

[7] Shan Z , Sun T, Huang H, Chen S, Chen L, Luo C et al. Association between microbiota-dependent metabolite trimethylamine-*N*-oxide and type 2 diabetes. Am J Clin Nutr. 2017;106(3):888–94. 28724646 10.3945/ajcn.117.157107

[8] Zhang W-Q , Wang Y-J, Zhang A, Ding Y-J, Zhang X-N, Jia Q-J et al. TMA/TMAO in hypertension: novel horizons and potential therapies. J Cardiovasc Transl Res. 2021;14(6):1117–24. 33709384 10.1007/s12265-021-10115-x

[9] Flores-Guerrero JL , Osté MCJ, Baraldi PB, Connelly MA, Garcia E, Navis G et al. Association of circulating trimethylamine *N*-oxide and its dietary determinants with the risk of kidney graft failure: results of the TransplantLines Cohort Study. Nutrients. 2021;13(1):262. 33477634 10.3390/nu13010262 PMC7831477

[10] Tang WHW , Wang Z, Kennedy DJ, Wu Y, Buffa JA, Agatisa-Boyle B et al. Gut microbiota-dependent trimethylamine *N*-oxide (TMAO) pathway contributes to both development of renal insufficiency and mortality risk in chronic kidney disease. Circ Res. 2015;116(3):448–55. 25599331 10.1161/CIRCRESAHA.116.305360 PMC4312512

[11] Zhang P , Zou J-Z, Chen J, Tan X, Xiang F-F, Shen B et al. Association of trimethylamine *N*-oxide with cardiovascular and all-cause mortality in hemodialysis patients. Ren Fail. 2020;42(1):1004–14. 32985309 10.1080/0886022X.2020.1822868 PMC7534338

[12] Berger M , Kleber ME, Delgado GE, März W, Andreas M, Hellstern P et al. Trimethylamine N-oxide and adenosine diphosphate–induced platelet reactivity are independent risk factors for cardiovascular and all-cause mortality. Circ Res. 2020;126(5):660–2. 31958034 10.1161/CIRCRESAHA.119.316214

[13] Winther SA , Ollgaard JC, Hansen TW, von Scholten BJ, Reinhard H, Ahluwalia TS et al. Plasma trimethylamine N-oxide and its metabolic precursors and risk of mortality, cardiovascular and renal disease in individuals with type 2-diabetes and albuminuria. PLoS One. 2021;16(3):e0244402. 33657115 10.1371/journal.pone.0244402 PMC7928450

[14] Flores-Guerrero JL , van Dijk PR, Connelly MA, Garcia E, Bilo HJG, Navis G et al. Circulating trimethylamine N-oxide is associated with increased risk of cardiovascular mortality in type-2 diabetes: results from a Dutch diabetes cohort (ZODIAC-59). J Clin Med. 2021;10(11):2269. 34073908 10.3390/jcm10112269 PMC8197378

[15] Hou L , Zhang Y, Zheng D, Shi H, Zou C, Zhang H et al. Increasing trimethylamine N-oxide levels as a predictor of early neurological deterioration in patients with acute ischemic stroke. Neurol Res. 2020;42(2):153–8. 31928326 10.1080/01616412.2019.1710416

[16] Chen M-l , Yi L, Zhang Y, Zhou X, Ran L, Yang J et al. Resveratrol attenuates trimethylamine-*N*-oxide (TMAO)-induced atherosclerosis by regulating TMAO synthesis and bile acid metabolism via remodeling of the gut microbiota. mBio. 2016;7(2):e02210–15. 27048804 10.1128/mBio.02210-15 PMC4817264

[17] Barrea L , Annunziata G, Muscogiuri G, Di Somma C, Laudisio D, Maisto M et al. Trimethylamine-N-oxide (TMAO) as novel potential biomarker of early predictors of metabolic syndrome. Nutrients. 2018;10(12):1971. 10.3390/nu10121971 PMC6316855 30551613

[18] Wilson A , Teft WA, Morse BL, Choi Y-H, Woolsey S, DeGorter MK et al. Trimethylamine-*N*-oxide: a novel biomarker for the identification of inflammatory bowel disease. Dig Dis Sci. 2015;60(12):3620–30. 26160437 10.1007/s10620-015-3797-3

[19] Chhibber-Goel J , Singhal V, Parakh N, Bhargava B, Sharma A. The metabolite trimethylamine-N-oxide is an emergent biomarker of human health. Curr Med Chem. 2017;24(36):3942–53. 27573063 10.2174/0929867323666160830104025

[20] Aromataris E , Fernandez R, Godfrey CM, Holly C, Khalil H, Tungpunkom P. Summarizing systematic reviews: methodological development, conduct and reporting of an umbrella review approach. Int J Evid Based Healthc. 2015;13(3):132–40. 26360830 10.1097/XEB.0000000000000055

[21] Dehghan P , Farhangi MA, Nikniaz L, Nikniaz Z, Asghari-Jafarabadi M. Gut microbiota-derived metabolite trimethylamine N-oxide (TMAO) potentially increases the risk of obesity in adults: an exploratory systematic review and dose-response meta- analysis. Obes Rev. 2020;21(5):e12993. 32017391 10.1111/obr.12993

[22] Farhangi MA , Vajdi M, Asghari-Jafarabadi M. Gut microbiota-associated metabolite trimethylamine *N*-oxide and the risk of stroke: a systematic review and dose–response meta-analysis. Nutr J. 2020;19(1):76. 32731904 10.1186/s12937-020-00592-2 PMC7393891

[23] Zhuang R , Ge X, Han L, Yu P, Gong X, Meng Q et al. Gut microbe–generated metabolite trimethylamine *N*-oxide and the risk of diabetes: a systematic review and dose-response meta-analysis. Obes Rev. 2019;20(6):883–94. 30868721 10.1111/obr.12843

[24] Abbasalizad Farhangi M , Vajdi M. Gut microbiota–associated trimethylamine *N*-oxide and increased cardiometabolic risk in adults: a systematic review and dose-response meta-analysis. Nutr Rev. 2021;79(9):1022–42. 33270896 10.1093/nutrit/nuaa111

[25] Heianza Y , Ma W, Manson JE, Rexrode KM, Qi L. Gut microbiota metabolites and risk of major adverse cardiovascular disease events and death: a systematic review and meta-analysis of prospective studies. J Am Heart Assoc. 2017;6(7):e004947. 28663251 10.1161/JAHA.116.004947 PMC5586261

[26] Stroup DF , Berlin JA, Morton SC, Olkin I, Williamson GD, Rennie D et al. Meta-analysis of observational studies in epidemiology: a proposal for reporting. JAMA. 2000;283(15):2008–12. 10789670 10.1001/jama.283.15.2008

[27] Duan H , Deng T, Chen Y, Zhao Z, Wen Y, Chen Y, Li X, Zeng G. Association between vasectomy and risk of testicular cancer: A systematic review and meta-analysis. PLoS One. 2018;13(3):e0194606. 29566037 10.1371/journal.pone.0194606 PMC5864054

[28] DerSimonian R , Laird N. Meta-analysis in clinical trials revisited. Contemp Clin Trials. 2015;45(Pt A):139–45. 26343745 10.1016/j.cct.2015.09.002 PMC4639420

[29] Higgins JPT , Thompson SG, Spiegelhalter DJ. A re-evaluation of random-effects meta-analysis. J R Stat Soc Ser A Stat Soc. 2009;172(1):137–59. 10.1111/j.1467-985X.2008.00552.x PMC2667312 19381330

[30] Higgins JPT . Commentary: heterogeneity in meta-analysis should be expected and appropriately quantified. Int J Epidemiol. 2008;37(5):1158–60. 18832388 10.1093/ije/dyn204

[31] Egger M , Davey Smith G, Schneider M, Minder C. Bias in meta-analysis detected by a simple, graphical test. BMJ. 1997;315(7109):629–34. 9310563 10.1136/bmj.315.7109.629 PMC2127453

[32] Ioannidis JP . Excess significance bias in the literature on brain volume abnormalities. Arch Gen Psychiatry. 2011;68(8):773–80. 21464342 10.1001/archgenpsychiatry.2011.28

[33] Kavvoura FK , McQueen MB, Khoury MJ, Tanzi RE, Bertram L, Ioannidis JP. Evaluation of the potential excess of statistically significant findings in published genetic association studies: application to Alzheimer's disease. Am J Epidemiol. 2008;168(8):855–65. 18779388 10.1093/aje/kwn206 PMC3695656

[34] Ioannidis JPA , Trikalinos TA. An exploratory test for an excess of significant findings. Clin Trials. 2007;4(3):245–53. 17715249 10.1177/1740774507079441

[35] Li X , Meng X, Timofeeva M, Tzoulaki I, Tsilidis KK, Ioannidis JP et al. Serum uric acid levels and multiple health outcomes: umbrella review of evidence from observational studies, randomised controlled trials, and Mendelian randomisation studies. BMJ. 2017;357:j2376. 28592419 10.1136/bmj.j2376 PMC5461476

[36] Kalliala I , Markozannes G, Gunter MJ, Paraskevaidis E, Gabra H, Mitra A et al. Obesity and gynaecological and obstetric conditions: umbrella review of the literature. BMJ. 2017;359:j4511. 29074629 10.1136/bmj.j4511 PMC5656976

[37] Kyrgiou M , Kalliala I, Markozannes G, Gunter MJ, Paraskevaidis E, Gabra H et al. Adiposity and cancer at major anatomical sites: umbrella review of the literature. BMJ. 2017;356:j477. 28246088 10.1136/bmj.j477 PMC5421437

[38] Schiattarella GG , Sannino A, Toscano E, Giugliano G, Gargiulo G, Franzone A et al. Gut microbe-generated metabolite trimethylamine-N-oxide as cardiovascular risk biomarker: a systematic review and dose-response meta-analysis. Eur Heart J. 2017;38(39):2948–56. 29020409 10.1093/eurheartj/ehx342

[39] Qi J , You T, Li J, Pan T, Xiang L, Han Y et al. Circulating trimethylamine N-oxide and the risk of cardiovascular diseases: a systematic review and meta-analysis of 11 prospective cohort studies. J Cell Mol Med. 2018;22(1):185–94. 28782886 10.1111/jcmm.13307 PMC5742728

[40] Farhangi MA . Gut microbiota-dependent trimethylamine N-oxide and all-cause mortality: findings from an updated systematic review and meta-analysis. Nutrition. 2020;78:110856. 32592979 10.1016/j.nut.2020.110856

[41] Farhangi MA , Vajdi M. Novel findings of the association between gut microbiota–derived metabolite trimethylamine *N*-oxide and inflammation: results from a systematic review and dose-response meta-analysis. Crit Rev Food Sci Nutr. 2020;60(16):2801–23. 32462890 10.1080/10408398.2020.1770199

[42] Ge X , Zheng L, Zhuang R, Yu P, Xu Z, Liu G et al. The gut microbial metabolite trimethylamine *N*-oxide and hypertension risk: a systematic review and dose–response meta-analysis. Adv Nutr. 2020;11(1):66–76. 31269204 10.1093/advances/nmz064 PMC7442397

[43] Li W , Huang A, Zhu H, Liu X, Huang X, Huang Y et al. Gut microbiota-derived trimethylamine *N*-oxide is associated with poor prognosis in patients with heart failure. Med J Aust. 2020;213(8):374–9. 32959366 10.5694/mja2.50781

[44] Yao M-E , Liao P-D, Zhao X-J, Wang L. Trimethylamine-N-oxide has prognostic value in coronary heart disease: a meta-analysis and dose-response analysis. BMC Cardiovasc Disord. 2020;20(1):7. 31918665 10.1186/s12872-019-01310-5 PMC6953212

[45] Guasti L , Galliazzo S, Molaro M, Visconti E, Pennella B, Gaudio GV et al. TMAO as a biomarker of cardiovascular events: a systematic review and meta-analysis. Intern Emerg Med. 2021;16(1):201–7. 32779113 10.1007/s11739-020-02470-5

[46] Yang W-T , Yang R, Zhao Q, Li X-D, Wang Y-T. A systematic review and meta-analysis of the gut microbiota-dependent metabolite trimethylamine N-oxide with the incidence of atrial fibrillation. Ann Palliat Med. 2021;10(11):11512–23. 34872276 10.21037/apm-21-2763

[47] Zeng Y , Guo M, Fang X, Teng F, Tan X, Li X et al. Gut microbiota-derived trimethylamine N-oxide and kidney function: a systematic review and meta-analysis. Adv Nutr. 2021;12(4):1286–304. 33751019 10.1093/advances/nmab010 PMC8321840

[48] Tang WHW , Wang Z, Levison BS, Koeth RA, Britt EB, Fu X et al. Intestinal microbial metabolism of phosphatidylcholine and cardiovascular risk. N Engl J Med. 2013;368(17):1575–84. 23614584 10.1056/NEJMoa1109400 PMC3701945

[49] Trøseid M , Ueland T, Hov JR, Svardal A, Gregersen I, Dahl CP et al. Microbiota-dependent metabolite trimethylamine-N-oxide is associated with disease severity and survival of patients with chronic heart failure. J Intern Med. 2015;277(6):717–26. 25382824 10.1111/joim.12328

[50] Tang WHW , Wang Z, Shrestha K, Borowski AG, Wu Y, Troughton RW et al. Intestinal microbiota-dependent phosphatidylcholine metabolites, diastolic dysfunction, and adverse clinical outcomes in chronic systolic heart failure. J Card Fail. 2015;21(2):91–6. 25459686 10.1016/j.cardfail.2014.11.006 PMC4312712

[51] Lever M , George PM, Slow S, Bellamy D, Young JM, Ho M et al. Betaine and trimethylamine-*N*-oxide as predictors of cardiovascular outcomes show different patterns in diabetes mellitus: an observational study. PLoS One. 2014;9(12):e114969. 25493436 10.1371/journal.pone.0114969 PMC4262445

[52] Kaysen GA , Johansen KL, Chertow GM, Dalrymple LS, Kornak J, Grimes B et al. Associations of trimethylamine *N*-oxide with nutritional and inflammatory biomarkers and cardiovascular outcomes in patients new to dialysis. J Ren Nutr. 2015;25(4):351–6. 25802017 10.1053/j.jrn.2015.02.006 PMC4469547

[53] Stubbs JR , House JA, Ocque AJ, Zhang S, Johnson C, Kimber C et al. Serum trimethylamine-*N*-oxide is elevated in CKD and correlates with coronary atherosclerosis burden. J Am Soc Nephrol. 2016;27(1):305–13. 26229137 10.1681/ASN.2014111063 PMC4696571

[54] Skagen K , Trøseid M, Ueland T, Holm S, Abbas A, Gregersen I et al. The carnitine-butyrobetaine-trimethylamine-N-oxide pathway and its association with cardiovascular mortality in patients with carotid atherosclerosis. Atherosclerosis. 2016;247:64–9. 26868510 10.1016/j.atherosclerosis.2016.01.033

[55] Senthong V , Wang Z, Li XS, Fan Y, Wu Y, Tang WHW et al. Intestinal microbiota-generated metabolite trimethylamine-*N*-oxide and 5-year mortality risk in stable coronary artery disease: the contributory role of intestinal microbiota in a COURAGE-like patient cohort. J Am Heart Assoc. 2016;5(6):e002816. 27287696 10.1161/JAHA.115.002816 PMC4937244

[56] Shafi T , Powe NR, Meyer TW, Hwang S, Hai X, Melamed ML et al. Trimethylamine *N*-oxide and cardiovascular events in hemodialysis patients. J Am Soc Nephrol. 2017;28(1):321–31. 27436853 10.1681/ASN.2016030374 PMC5198291

[57] Ottiger M , Nickler M, Steuer C, Odermatt J, Huber A, Christ-Crain M et al. Trimethylamine-N-oxide (TMAO) predicts fatal outcomes in community-acquired pneumonia patients without evident coronary artery disease. Eur J Intern Med. 2016;36:67–73. 27567042 10.1016/j.ejim.2016.08.017

[58] Senthong V , Wang Z, Fan Y, Wu Y, Hazen SL, Tang WHW. Trimethylamine *N*-oxide and mortality risk in patients with peripheral artery disease. J Am Heart Assoc. 2016;5(10):e004237. 27792653 10.1161/JAHA.116.004237 PMC5121520

[59] Suzuki T , Heaney LM, Jones DJL, Ng LL. Trimethylamine *N*-oxide and risk stratification after acute myocardial infarction. Clin Chem. 2017;63(1):420–8. 28062632 10.1373/clinchem.2016.264853

[60] Schuett K , Kleber ME, Scharnagl H, Lorkowski S, März W, Niessner A et al. Trimethylamine-N-oxide and heart failure with reduced versus preserved ejection fraction. J Am Coll Cardiol. 2017;70(25):3202–4. 29268932 10.1016/j.jacc.2017.10.064

[61] Suzuki T , Yazaki Y, Voors AA, Jones DJL, Chan DCS, Anker SD et al. Association with outcomes and response to treatment of trimethylamine N-oxide in heart failure: results from BIOSTAT-CHF. Eur J Heart Fail. 2019;21(7):877–86. 30370976 10.1002/ejhf.1338

[62] Reiner MF , Müller D, Gobbato S, Stalder O, Limacher A, Bonetti NR et al. Gut microbiota-dependent trimethylamine-*N*-oxide (TMAO) shows a U-shaped association with mortality but not with recurrent venous thromboembolism. Thromb Res. 2019;174:40–7. 30553164 10.1016/j.thromres.2018.12.011

[63] Salzano A , Israr MZ, Yazaki Y, Heaney LM, Kanagala P, Singh A et al. Combined use of trimethylamine N-oxide with BNP for risk stratification in heart failure with preserved ejection fraction: findings from the DIAMONDHFpEF study. Eur J Prev Cardiol. 2020;27(19):2159–62. 31412713 10.1177/2047487319870355

[64] Zhang J , Wang L, Cai J, Lei A, Liu C, Lin R et al. Gut microbial metabolite TMAO portends prognosis in acute ischemic stroke. J Neuroimmunol. 2021;354:577526. 33647820 10.1016/j.jneuroim.2021.577526

[65] Tang WHW , Wang Z, Fan Y, Levison B, Hazen JE, Donahue LM et al. Prognostic value of elevated levels of intestinal microbe-generated metabolite trimethylamine-*N*-oxide in patients with heart failure: refining the gut hypothesis. J Am Coll Cardiol. 2014;64(18):1908–14. 25444145 10.1016/j.jacc.2014.02.617 PMC4254529

[66] Tang WHW , Wang Z, Li XS, Fan Y, Li DS, Wu Y et al. Increased trimethylamine *N*-oxide portends high mortality risk independent of glycemic control in patients with type 2 diabetes mellitus. Clin Chem. 2017;63(1):297–306. 27864387 10.1373/clinchem.2016.263640 PMC5659115

[67] Winther SA , Øllgaard JC, Tofte N, Tarnow L, Wang Z, Ahluwalia TS et al. Utility of plasma concentration of trimethylamine N-oxide in predicting cardiovascular and renal complications in individuals with type 1 diabetes. Diabetes Care. 2019;42(8):1512–20. 31123156 10.2337/dc19-0048 PMC7082641

[68] Croyal M , Saulnier P-J, Aguesse A, Gand E, Ragot S, Roussel R et al. Plasma trimethylamine N-oxide and risk of cardiovascular events in patients with type 2 diabetes. J Clin Endocrinol Metab. 2020;105(7):2371–80. 10.1210/clinem/dgaa188 32301490

[69] Stubbs JR , Stedman MR, Liu S, Long J, Franchetti Y, West RE 3rd et al. Trimethylamine *N*-oxide and cardiovascular outcomes in patients with ESKD receiving maintenance hemodialysis. Clin J Am Soc Nephrol. 2019;14(2):261–7. 30665924 10.2215/CJN.06190518 PMC6390920

[70] Zhou X , Jin M, Liu L, Yu Z, Lu X, Zhang H. Trimethylamine N-oxide and cardiovascular outcomes in patients with chronic heart failure after myocardial infarction. ESC Heart Fail. 2020;7(1):188–93. 31960610 10.1002/ehf2.12552 PMC7083407

[71] Hochstrasser SR , Metzger K, Vincent AM, Becker C, Keller AKJ, Beck K et al. Trimethylamine-N-oxide (TMAO) predicts short- and long-term mortality and poor neurological outcome in out-of-hospital cardiac arrest patients. Clin Chem Lab Med. 2021;59(2):393–402. 10.1515/cclm-2020-0159 32866111

[72] Flores-Guerrero JL , Post A, van Dijk PR, Connelly MA, Garcia E, Navis G et al. Circulating trimethylamine-*N*-oxide is associated with all-cause mortality in subjects with nonalcoholic fatty liver disease. Liver Int. 2021;41(10):2371–82. 33993608 10.1111/liv.14963 PMC8518486

[73] Yazaki Y , Aizawa K, Israr MZ, Negishi K, Salzano A, Saitoh Y et al. Ethnic differences in association of outcomes with trimethylamine N-oxide in acute heart failure patients. ESC Heart Fail. 2020;7(5):2373–8. 32598563 10.1002/ehf2.12777 PMC7524106

[74] Ottiger M , Nickler M, Steuer C, Bernasconi L, Huber A, Christ-Crain M et al. Gut, microbiota-dependent trimethylamine-*N*-oxide is associated with long-term all-cause mortality in patients with exacerbated chronic obstructive pulmonary disease. Nutrition. 2018;45:135–41.e1. 28870405 10.1016/j.nut.2017.07.001

[75] Wang Z , Tang WHW, Buffa JA, Fu X, Britt EB, Koeth RA et al. Prognostic value of choline and betaine depends on intestinal microbiota-generated metabolite trimethylamine-*N*-oxide. Eur Heart J. 2014;35(14):904–10. 24497336 10.1093/eurheartj/ehu002 PMC3977137

[76] Missailidis C , Hallqvist J, Qureshi AR, Barany P, Heimburger O, Lindholm B et al. Serum trimethylamine-*N*-oxide is strongly related to renal function and predicts outcome in chronic kidney disease. PLoS One. 2016;11(1):e0141738. 26751065 10.1371/journal.pone.0141738 PMC4709190

[77] Suzuki T , Heaney LM, Bhandari SS, Jones DJ, Ng LL. Trimethylamine *N*-oxide and prognosis in acute heart failure. Heart. 2016;102(11):841–8. 26869641 10.1136/heartjnl-2015-308826

[78] Kim RB , Morse BL, Djurdjev O, Tang M, Muirhead N, Barrett B et al. Advanced chronic kidney disease populations have elevated trimethylamine N-oxide levels associated with increased cardiovascular events. Kidney Int. 2016;89(5):1144–52. 27083288 10.1016/j.kint.2016.01.014

[79] Robinson-Cohen C , Newitt R, Shen DD, Rettie AE, Kestenbaum BR, Himmelfarb J et al. Association of FMO3 variants and trimethylamine N-oxide concentration, disease progression, and mortality in CKD patients. PLoS One. 2016;11(8):e0161074. 27513517 10.1371/journal.pone.0161074 PMC4981377

[80] Matsuzawa Y , Nakahashi H, Konishi M, Sato R, Kawashima C, Kikuchi S et al. Microbiota-derived trimethylamine N-oxide predicts cardiovascular risk after STEMI. Sci Rep. 2019;9(1):11647. 31406181 10.1038/s41598-019-48246-6 PMC6690996

[81] Nam HS , Ha J, Ji D, Kwon I, Lee HS, Han M et al. Elevation of the gut microbiota metabolite trimethylamine N-oxide predicts stroke outcome. J Stroke. 2019;21(3):350–2. 31590480 10.5853/jos.2019.00850 PMC6780019

[82] Xu K-Zu , Lin LM, Ying Wu, Xu J-H, Wu M-F, Do C. Relationship between plasma trimethylamine-N-oxide levels and complication risk in patients with acute myocardial infarction. Chinese J Arterioscler. 2018;26:497–502.

[83] Gencer B , Li XS, Gurmu Y, Bonaca MP, Morrow DA, Cohen M et al. Gut microbiota-dependent trimethylamine N-oxide and cardiovascular outcomes in patients with prior myocardial infarction: a nested case control study from the PEGASUS-TIMI 54 trial. J Am Heart Assoc. 2020;9(10):e015331. 32366163 10.1161/JAHA.119.015331 PMC7660879

[84] Zheng Y , Tang Z, You L, Wu Y, Liu J, Xue J. Trimethylamine-*N*-oxide is an independent risk factor for hospitalization events in patients receiving maintenance hemodialysis. Ren Fail. 2020;42(1):580–6. 32576072 10.1080/0886022X.2020.1781170 PMC7946050

[85] Kinugasa Y , Nakamura K, Kamitani H, Hirai M, Yanagihara K, Kato M et al. Trimethylamine N-oxide and outcomes in patients hospitalized with acute heart failure and preserved ejection fraction. ESC Heart Fail. 2021;8(3):2103–10. 33734604 10.1002/ehf2.13290 PMC8120352

[86] Liu X , Xie Z, Sun M, Wang X, Li J, Cui J et al. Plasma trimethylamine N-oxide is associated with vulnerable plaque characteristics in CAD patients as assessed by optical coherence tomography. Int J Cardiol. 2018;265:18–23. 29729869 10.1016/j.ijcard.2018.04.126

[87] Roy S , Yuzefpolskaya M, Nandakumar R, Colombo PC, Demmer RT. Plasma trimethylamine-N-oxide and impaired glucose regulation: results from the Oral Infections, Glucose Intolerance and Insulin Resistance Study (ORIGINS). PLoS One. 2020;15(1):e0227482. 31940332 10.1371/journal.pone.0227482 PMC6961885

[88] Zhu C , Li G, Lv Z, Li J, Wang X, Kang J et al. Association of plasma trimethylamine-N-oxide levels with post-stroke cognitive impairment: a 1-year longitudinal study. Neurolog Sci. 2020;41(1):57–63. 10.1007/s10072-019-04040-w 31420758

[89] Svingen GFT , Zuo H, Ueland PM, Seifert R, Loland KH, Pedersen ER et al. Increased plasma trimethylamine-*N*-oxide is associated with incident atrial fibrillation. Int J Cardiol. 2018;267:100–6. 29957250 10.1016/j.ijcard.2018.04.128

[90] Mafune A , Iwamoto T, Tsutsumi Y, Nakashima A, Yamamoto I, Yokoyama K et al. Associations among serum trimethylamine-N-oxide (TMAO) levels, kidney function and infarcted coronary artery number in patients undergoing cardiovascular surgery: a cross-sectional study. Clin Exp Nephrol. 2016;20(5):731–9. 26676906 10.1007/s10157-015-1207-y PMC5050242

[91] Zheng L , Zheng J, Xie Y, Li Z, Guo X, Sun G et al. Serum gut microbe-dependent trimethylamine N-oxide improves the prediction of future cardiovascular disease in a community-based general population. Atherosclerosis. 2019;280:126–31. 30508691 10.1016/j.atherosclerosis.2018.11.010

[92] Yu D , Shu X-O, Rivera ES, Zhang X, Cai Q, Calcutt MW et al. Urinary levels of trimethylamine-N-oxide and incident coronary heart disease: a prospective investigation among urban Chinese adults. J Am Heart Assoc. 2019;8(1):e010606. 30606084 10.1161/JAHA.118.010606 PMC6405718

[93] Schneider C , Okun JG, Schwarz KV, Hauke J, Zorn M, Nürnberg C et al. Trimethylamine-N-oxide is elevated in the acute phase after ischaemic stroke and decreases within the first days. Eur J Neurol. 2020;27(8):1596–603. 32282978 10.1111/ene.14253

[94] Tang WHW , Li XS, Wu Y, Wang Z, Khaw K-T, Wareham NJ et al. Plasma trimethylamine *N*-oxide (TMAO) levels predict future risk of coronary artery disease in apparently healthy individuals in the EPIC-Norfolk prospective population study. Am Heart J. 2021;236:80–6. 33626384 10.1016/j.ahj.2021.01.020 PMC8085024

[95] Lee Y , Nemet I, Wang Z, Lai HTM, de Oliveira Otto MC, Lemaitre RN et al. Longitudinal plasma measures of trimethylamine N-oxide and risk of atherosclerotic cardiovascular disease events in community-based older adults. J Am Heart Assoc. 2021;10(17):e020646. 34398665 10.1161/JAHA.120.020646 PMC8649305

[96] Senthong V , Kiatchoosakun S, Wongvipaporn C, Phetcharaburanin J, Tatsanavivat P, Sritara P et al. Gut microbiota-generated metabolite, trimethylamine-*N*-oxide, and subclinical myocardial damage: a multicenter study from Thailand. Sci Rep. 2021;11(1):14963. 34294762 10.1038/s41598-021-93803-7 PMC8298599

[97] Guasch-Ferré M , Hu FB, Ruiz-Canela M, Bulló M, Toledo E, Wang DD et al. Plasma metabolites from choline pathway and risk of cardiovascular disease in the PREDIMED (Prevention with Mediterranean Diet) study. J Am Heart Assoc. 2017;6(11):e006524. 10.1161/JAHA.117.006524 PMC5721752 29080862

[98] Nie J , Xie L, Zhao B-x, Li Y, Qiu B, Zhu F et al. Serum trimethylamine N-oxide concentration is positively associated with first stroke in hypertensive patients. Stroke. 2018;49(9):2021–8. 30354996 10.1161/STROKEAHA.118.021997

[99] Wu C , Xue F, Lian Y, Zhang J, Wu D, Xie N et al. Relationship between elevated plasma trimethylamine N-oxide levels and increased stroke injury. Neurology. 2020;94(7):e667–77. 31907287 10.1212/WNL.0000000000008862

[100] Sun T , Zhang Y, Yin J, Peng X, Zhou L, Huang S et al. Association of gut microbiota-dependent metabolite trimethylamine N-oxide with first ischemic stroke. J Atheroscler Thromb. 2021;28(4):320–8. 32641646 10.5551/jat.55962 PMC8147013

[101] Obeid R , Awwad HM, Rabagny Y, Graeber S, Herrmann W, Geisel J. Plasma trimethylamine *N*-oxide concentration is associated with choline, phospholipids, and methyl metabolism. Am J Clin Nutr. 2016;103(3):703–11. 26864355 10.3945/ajcn.115.121269

[102] Fu D , Shen J, Li W, Wang Y, Zhong Z, Ye H et al. Elevated serum trimethylamine N-oxide levels are associated with mortality in male patients on peritoneal dialysis. Blood Purif. 2021;50(6):837–47. 33596582 10.1159/000512962

[103] Mente A , Chalcraft K, Ak H, Davis AD, Lonn E, Miller R et al. The relationship between trimethylamine-N-oxide and prevalent cardiovascular disease in a multiethnic population living in Canada. Can J Cardiol. 2015;31(9):1189–94. 26239008 10.1016/j.cjca.2015.06.016

[104] Papandreou C , Bulló M, Zheng Y, Ruiz-Canela M, Yu E, Guasch-Ferré M et al. Plasma trimethylamine-N-oxide and related metabolites are associated with type 2 diabetes risk in the Prevención con Dieta Mediterránea (PREDIMED) trial. Am J Clin Nutr. 2018;108(1):163–73. 29982310 10.1093/ajcn/nqy058 PMC6862602

[105] Lemaitre RN , Jensen PN, Wang Z, Fretts AM, McKnight B, Nemet I et al. Association of trimethylamine *N*-oxide and related metabolites in plasma and incident type 2 diabetes: the Cardiovascular Health Study. JAMA Netw Open. 2021;4(8):e2122844. 34448864 10.1001/jamanetworkopen.2021.22844 PMC8397925

[106] Bae S , Ulrich CM, Neuhouser ML, Malysheva O, Bailey LB, Xiao L et al. Plasma choline metabolites and colorectal cancer risk in the Women's Health Initiative Observational Study. Cancer Res. 2014;74(24):7442–52. 25336191 10.1158/0008-5472.CAN-14-1835 PMC4268282

[107] Guertin KA , Li XS, Graubard BI, Albanes D, Weinstein SJ, Goedert JJ et al. Serum trimethylamine N-oxide, carnitine, choline, and betaine in relation to colorectal cancer risk in the Alpha Tocopherol, Beta Carotene Cancer Prevention Study. Cancer Epidemiol Biomarkers Prev. 2017;26(6):945–52. 28077427 10.1158/1055-9965.EPI-16-0948 PMC5608021

[108] Liu X , Liu H, Yuan C, Zhang Y, Wang W, Hu S et al. Preoperative serum TMAO level is a new prognostic marker for colorectal cancer. Biomark Med. 2017;11(5):443–7. 28621609 10.2217/bmm-2016-0262

[109] Mondul AM , Moore SC, Weinstein SJ, Karoly ED, Sampson JN, Albanes D. Metabolomic analysis of prostate cancer risk in a prospective cohort: the alpha-tocopherol, beta-carotene cancer prevention (ATBC) study. Int J Cancer. 2015;137(9):2124–32. 25904191 10.1002/ijc.29576 PMC4537663

[110] Liu Z-Y , Tan X-Y, Li Q-J, Liao G-C, Fang A-P, Zhang D-M et al. Trimethylamine N-oxide, a gut microbiota-dependent metabolite of choline, is positively associated with the risk of primary liver cancer: a case-control study. Nutr Metab. 2018;15(1):81. 10.1186/s12986-018-0319-2 PMC6245753 30479648

[111] Huang JY , Luu HN, Butler LM, Midttun O, Ulvik A, Wang R et al. A prospective evaluation of serum methionine-related metabolites in relation to pancreatic cancer risk in two prospective cohort studies. Int J Cancer. 2020;147(7):1917–27. 32222976 10.1002/ijc.32994 PMC11537248

[112] Haghikia A , Li XS, Liman TG, Bledau N, Schmidt D, Zimmermann F et al. Gut microbiota–dependent trimethylamine *N*-oxide predicts risk of cardiovascular events in patients with stroke and is related to proinflammatory monocytes. Arterioscler Thromb Vasc Biol. 2018;38(9):2225–35. 29976769 10.1161/ATVBAHA.118.311023 PMC6202215

[113] Randrianarisoa E , Lehn-Stefan A, Wang X, Hoene M, Peter A, Heinzmann SS et al. Relationship of serum trimethylamine N-oxide (TMAO) levels with early atherosclerosis in humans. Sci Rep. 2016;6:26745. 27228955 10.1038/srep26745 PMC4882652

[114] Krüger R , Merz B, Rist MJ, Ferrario PG, Bub A, Kulling SE et al. Associations of current diet with plasma and urine TMAO in the KarMeN study: direct and indirect contributions. Mol Nutr Food Res. 2017;61(11):1700363. 10.1002/mnfr.201700363 28755411

[115] Meyer KA , Benton TZ, Bennett BJ, Jacobs DR Jr, Lloyd-Jones DM, Gross MD et al. Microbiota-dependent metabolite trimethylamine N-oxide and coronary artery calcium in the Coronary Artery Risk Development in Young Adults Study (CARDIA). J Am Heart Assoc. 2016;5(10):e003970. 27792658 10.1161/JAHA.116.003970 PMC5121500

[116] Aslibekyan S , Irvin MR, Hidalgo BA, Perry RT, Jeyarajah EJ, Garcia E et al. Genome- and CD4^+^ T-cell methylome-wide association study of circulating trimethylamine-N-oxide in the Genetics of Lipid Lowering Drugs and Diet Network (GOLDN). J Nutr Intermed Metab. 2017;8:1–7. 28439531 10.1016/j.jnim.2017.03.002 PMC5400362

[117] Huang X , Li Z, Gao Z, Wang D, Li X, Li Y et al. Association between risk of preeclampsia and maternal plasma trimethylamine-N-oxide in second trimester and at the time of delivery. BMC Pregnancy Childbirth. 2020;20(1):302. 32429856 10.1186/s12884-020-02997-7 PMC7236207

[118] Liu W , Wang C, Xia Y, Xia W, Liu G, Ren C et al. Elevated plasma trimethylamine-*N*-oxide levels are associated with diabetic retinopathy. Acta Diabetol. 2021;58(2):221–9. 33064205 10.1007/s00592-020-01610-9 PMC7889550

[119] Liu Y , Guo Y-L, Meng S, Gao H, Sui L-J, Jin S et al. Gut microbiota–dependent trimethylamine N-oxide are related with hip fracture in postmenopausal women: a matched case-control study. Aging. 2020;12(11):10633–41. 32482913 10.18632/aging.103283 PMC7346070

[120] Chung SJ , Rim JH, Ji D, Lee S, Yoo HS, Jung JH et al. Gut microbiota-derived metabolite trimethylamine N-oxide as a biomarker in early Parkinson's disease. Nutrition. 2021;83:111090. 33418492 10.1016/j.nut.2020.111090

[121] Chen Y-m , Liu Y, Zhou R-f, Chen X-l, Wang C, Tan X-y et al. Associations of gut-flora-dependent metabolite trimethylamine-N-oxide, betaine and choline with non-alcoholic fatty liver disease in adults. Sci Rep. 2016;6(1):19076. 26743949 10.1038/srep19076 PMC4705470

[122] León-Mimila P , Villamil-Ramírez H, Li XS, Shih DM, Hui ST, Ocampo-Medina E et al. Trimethylamine N-oxide levels are associated with NASH in obese subjects with type 2 diabetes. Diabetes Metab. 2021;47(2):101183. 32791310 10.1016/j.diabet.2020.07.010 PMC8018562

[123] Wang Z , Zhao Y. Gut microbiota derived metabolites in cardiovascular health and disease. Protein Cell. 2018;9(5):416–31. 29725935 10.1007/s13238-018-0549-0 PMC5960473

[124] Jia J , Dou P, Gao M, Kong X, Li C, Liu Z et al. Assessment of causal direction between gut microbiota–dependent metabolites and cardiometabolic health: a bidirectional Mendelian randomization analysis. Diabetes. 2019;68(9):1747–55. 31167879 10.2337/db19-0153

[125] Yan Q , Gu Y, Li X, Yang W, Jia L, Chen C et al. Alterations of the gut microbiome in hypertension. Front Cell Infect Microbiol. 2017;7:381. 28884091 10.3389/fcimb.2017.00381 PMC5573791

[126] Jiang S , Shui Y, Cui Y, Tang C, Wang X, Qiu X et al. Gut microbiota dependent trimethylamine N-oxide aggravates angiotensin II–induced hypertension. Redox Biol. 2021;46:102115. 34474396 10.1016/j.redox.2021.102115 PMC8408632

[127] Ufnal M , Jazwiec R, Dadlez M, Drapala A, Sikora M, Skrzypecki J. Trimethylamine-N-oxide: a carnitine-derived metabolite that prolongs the hypertensive effect of angiotensin II in rats. Can J Cardiol. 2014;30(12):1700–5. 25475471 10.1016/j.cjca.2014.09.010

[128] Liu M , Han Q, Yang J. Trimethylamine-N-oxide (TMAO) increased aquaporin-2 expression in spontaneously hypertensive rats. Clin Exp Hypertens. 2019;41(4):312–22. 29985655 10.1080/10641963.2018.1481420

[129] Gao X , Tian Y, Randell E, Zhou H, Sun G. Unfavorable associations between serum trimethylamine N-oxide and L-carnitine levels with components of metabolic syndrome in the Newfoundland population. Front Endocrinol. 2019;10:168. 10.3389/fendo.2019.00168 PMC6443640 30972022

[130] Borges CC , Salles AF, Bringhenti I, Mandarim D, Aguila MB. Vitamin D deficiency increases lipogenesis and reduces beta-oxidation in the liver of diet-induced obese mice. J Nutr Sci Vitaminol (Tokyo). 2018;64(2):106–15. 29710028 10.3177/jnsv.64.106

[131] Gao X , Liu X, Xu J, Xue C, Xue Y, Wang Y. Dietary trimethylamine *N*-oxide exacerbates impaired glucose tolerance in mice fed a high fat diet. J Biosci Bioeng. 2014;118(4):476–81. 24721123 10.1016/j.jbiosc.2014.03.001

[132] Shih DM , Wang Z, Lee R, Meng Y, Che N, Charugundla S et al. Flavin containing monooxygenase 3 exerts broad effects on glucose and lipid metabolism and atherosclerosis. J Lipid Res. 2015;56(1):22–37. 25378658 10.1194/jlr.M051680 PMC4274068

[133] Li P , Zhong C, Li S, Sun T, Huang H, Chen X et al. Plasma concentration of trimethylamine-*N*-oxide and risk of gestational diabetes mellitus. Am J Clin Nutr. 2018;108(3):603–10. 30535087 10.1093/ajcn/nqy116 PMC6924263

[134] Huo X , Li J, Cao Y-F, Li S-N, Shao P, Leng J et al. Trimethylamine *N*-oxide metabolites in early pregnancy and risk of gestational diabetes: a nested case-control study. J Clin Endocrinol Metab. 2019;104(11):5529–39. 31373635 10.1210/jc.2019-00710 PMC6779108

[135] Gupta N , Buffa JA, Roberts AB, Sangwan N, Skye SM, Li L et al. Targeted inhibition of gut microbial trimethylamine N-oxide production reduces renal tubulointerstitial fibrosis and functional impairment in a murine model of chronic kidney disease. Arterioscler Thromb Vasc Biol. 2020;40(5):1239–55. 32212854 10.1161/ATVBAHA.120.314139 PMC7203662

[136] Wang P , Luo M-L, Song E, Zhou Z, Ma T, Wang J et al. Long noncoding RNA *lnc-TSI* inhibits renal fibrogenesis by negatively regulating the TGF-β/Smad3 pathway. Sci Transl Med. 2018;10(462):aat2039. 10.1126/scitranslmed.aat2039 30305452

[137] Zhang W , Miikeda A, Zuckerman J, Jia X, Charugundla S, Zhou Z et al. Inhibition of microbiota-dependent TMAO production attenuates chronic kidney disease in mice. Sci Rep. 2021;11(1):518. 33436815 10.1038/s41598-020-80063-0 PMC7804188

[138] Kalagi NA , Abbott KA, Alburikan KA, Alkofide HA, Stojanovski E, Garg ML. Modulation of circulating trimethylamine *N*-oxide concentrations by dietary supplements and pharmacological agents: a systematic review. Adv Nutr. 2019;10(5):876–87. 31073588 10.1093/advances/nmz012 PMC6743816

[139] Janeiro MH , Ramírez MJ, Milagro FI, Martínez JA, Solas M. Implication of trimethylamine N-oxide (TMAO) in disease: potential biomarker or new therapeutic target. Nutrients. 2018;10(10):1398. 10.3390/nu10101398 PMC6213249 30275434

[140] Simmonds M , Salanti G, McKenzie J, Elliott J; Living Systematic Review Network. Living systematic reviews: 3. Statistical methods for updating meta-analyses. J Clin Epidemiol. 2017;91:38–46. 28912004 10.1016/j.jclinepi.2017.08.008

