# Supplementary material for: Gut microbiota–derived metabolite trimethylamine-N-oxide and multiple health outcomes: an umbrella review and updated meta-analysis
Source: Am J Clin Nutr. 2022 Mar 28;116(1):230–43. doi: 10.1093/ajcn/nqac074 (PMC9257469; doi:10.1093/ajcn/nqac074)
Supplement: nqac074_Supplemental_File [file nqac074_supplemental_file.zip › Supplementary Figure 9-14.docx]

**On-line Supplementary Material**

Gut microbiota-derived metabolite Trimethylamine-N-oxide (TMAO) and multiple health outcomes: an umbrella review and updated meta-analysis

Li et al.

**Supplementary Figures 9-14:**

Supplementary Figure 9. Forest plot showing the risk of the effect of Trimethylamine N-oxide (TMAO) on cardiovascular disease (CVD) mortality.

Supplementary Figure 10. Forest plot showing the risk of the effect of Trimethylamine N-oxide (TMAO) on stroke.

Supplementary Figure 11. Forest plot showing the risk of the effect of Trimethylamine N-oxide (TMAO) on diabetes mellitus (DM)*.*

Supplementary Figure 12. Forest plot showing the risk of the effect of Trimethylamine N-oxide (TMAO) on diabetes mellitus (DM) (only included cohort studies that also adjusted for renal function)*.*

Supplementary Figure 13. Forest plot showing the risk of the effect of Trimethylamine N-oxide (TMAO) on gestational diabetes mellitus (GDM).

Supplementary Figure 14. Forest plot showing the risk of the effect of Trimethylamine N-oxide (TMAO) on colorectal cancer (CRC).


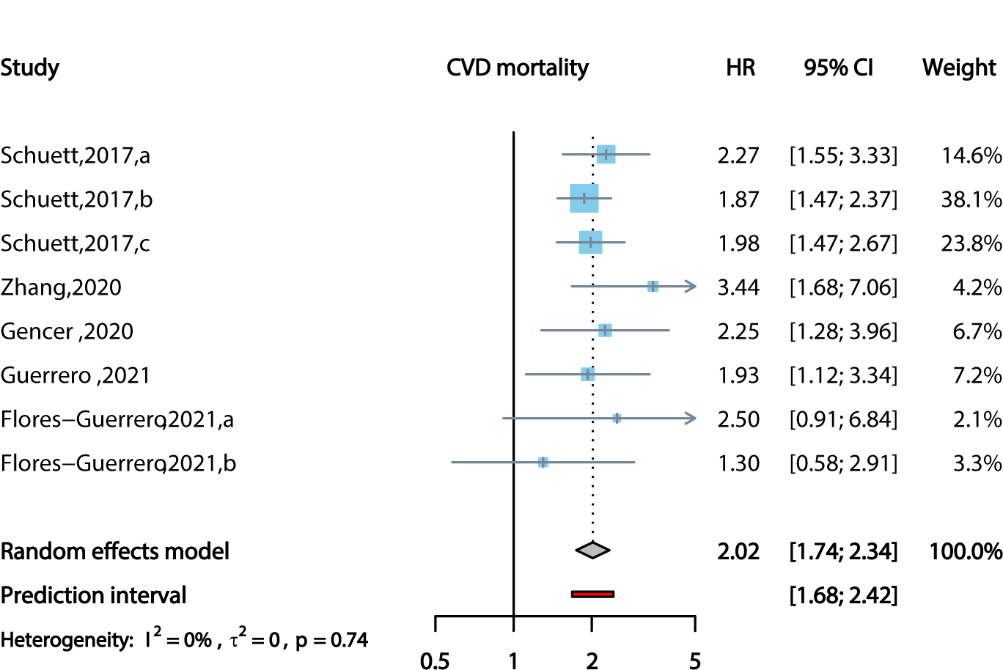


Supplementary Figure 9. Forest plot showing the risk of the effect of Trimethylamine N-oxide (TMAO) on cardiovascular disease (CVD) mortality. HR: hazard ratio. The diamond represents the pooled risk estimate. Interstudy heterogeneity was tested using the Cochran Q statistic(t^2^) at a significance level of *P*<0.10 and quantified by the *I^2^* statistic. An *I*^2^ value ≥50% is considered to indicate substantial heterogeneity. All results are presented as hazard ratio with 95% confidence intervals, using the Mantel-Haenszel method with a random-effects model.


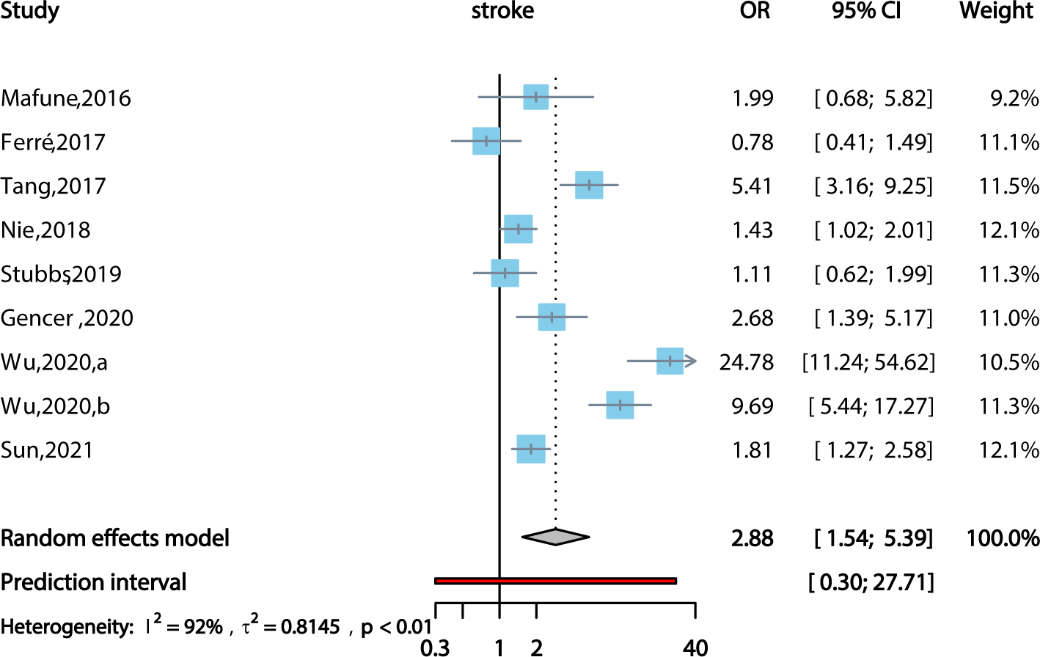


Supplementary Figure 10. Forest plot showing the risk of the effect of Trimethylamine N-oxide (TMAO) on stroke. OR: odds ratio. The diamond represents the pooled risk estimate. Interstudy heterogeneity was tested using the Cochran Q statistic(t^2^) at a significance level of *P*<0.10 and quantified by the *I^2^* statistic. An *I*^2^ value ≥50% is considered to indicate substantial heterogeneity. All results are presented as odds ratio with 95% confidence intervals, using the Mantel-Haenszel method with a random-effects model.


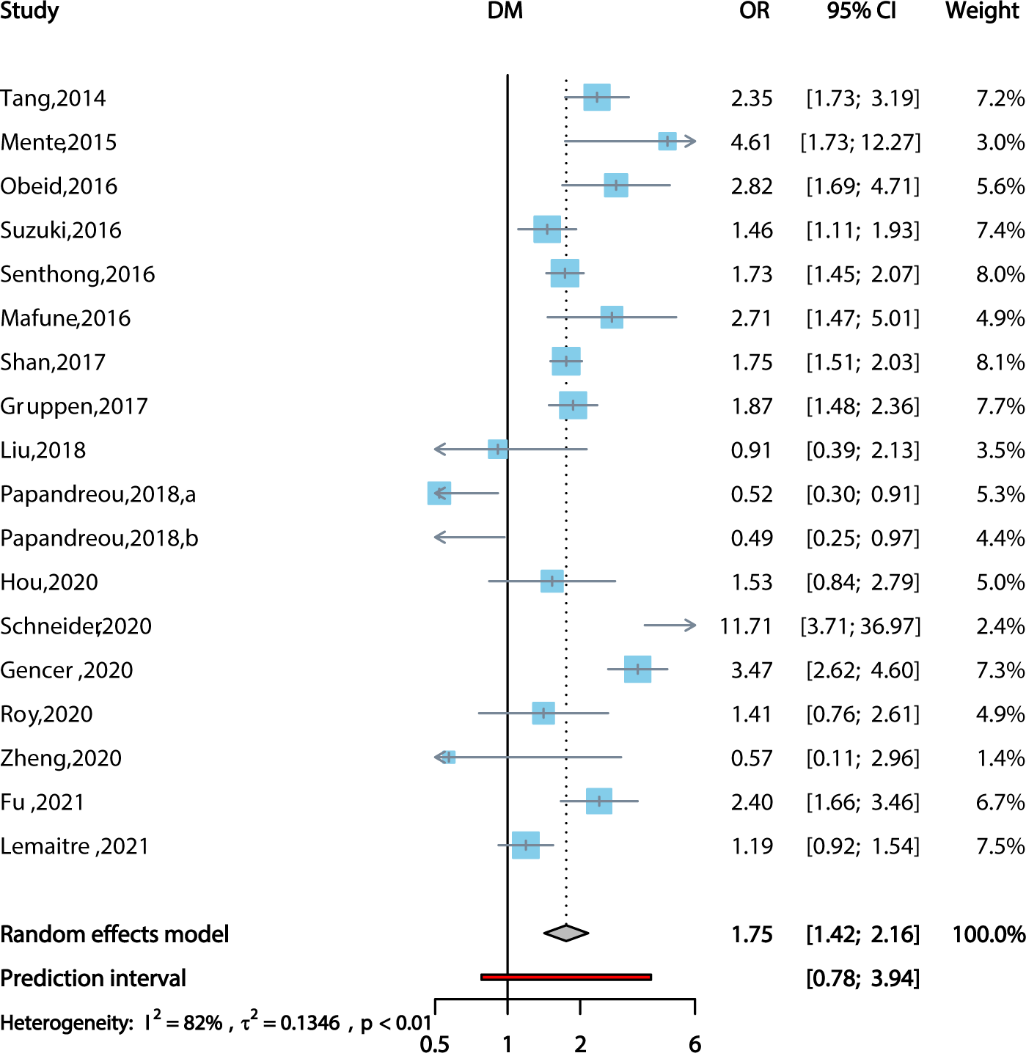


Supplementary Figure 11. Forest plot showing the risk of the effect of Trimethylamine N-oxide (TMAO) on diabetes mellitus (DM)*.* OR: odds ratio. The diamond represents the pooled risk estimate. Interstudy heterogeneity was tested using the Cochran Q statistic(t^2^) at a significance level of *P*<0.10 and quantified by the *I^2^* statistic. An *I*^2^ value ≥50% is considered to indicate substantial heterogeneity. All results are presented as odds ratio with 95% confidence intervals, using the Mantel-Haenszel method with a random-effects model.

*
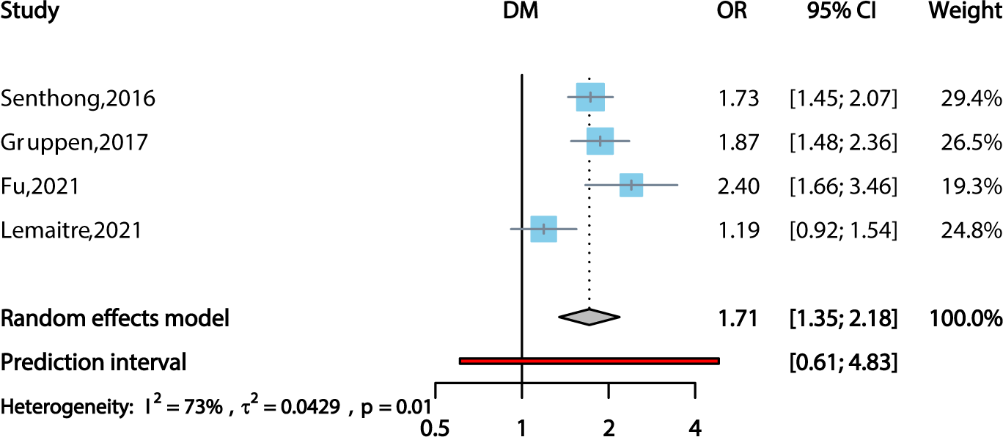
*

Supplementary Figure 12. Forest plot showing the risk of the effect of Trimethylamine N-oxide (TMAO) on diabetes mellitus (DM) (only included cohort studies that also adjusted for renal function)*.* The diamond represents the pooled risk estimate. Interstudy heterogeneity was tested using the Cochran Q statistic(t^2^) at a significance level of *P*<0.10 and quantified by the *I^2^* statistic. An *I*^2^ value ≥50% is considered to indicate substantial heterogeneity. All results are presented as odds ratio with 95% confidence intervals, using the Mantel-Haenszel method with a random-effects model.


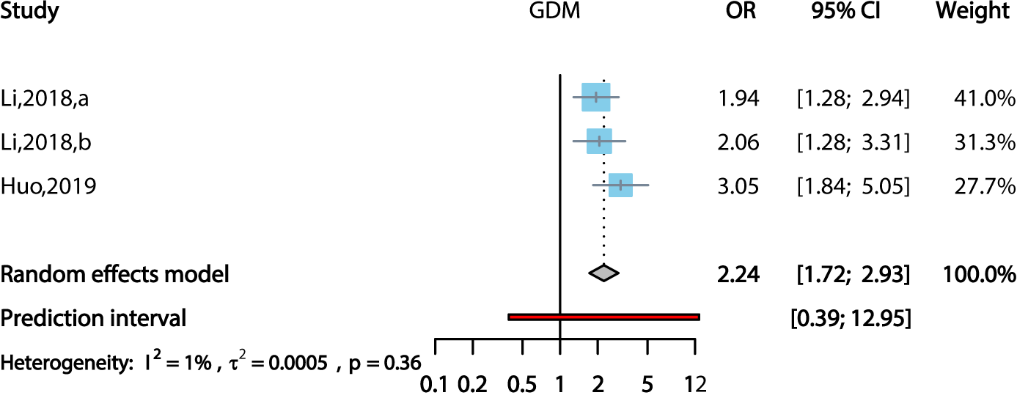


Supplementary Figure 13. Forest plot showing the risk of the effect of Trimethylamine N-oxide (TMAO) on gestational diabetes mellitus (GDM). OR: odds ratio. The diamond represents the pooled risk estimate. Interstudy heterogeneity was tested using the Cochran Q statistic(t^2^) at a significance level of *P*<0.10 and quantified by the *I^2^* statistic. An *I*^2^ value ≥50% is considered to indicate substantial heterogeneity. All results are presented as odds ratio with 95% confidence intervals, using the Mantel-Haenszel method with a random-effects model.


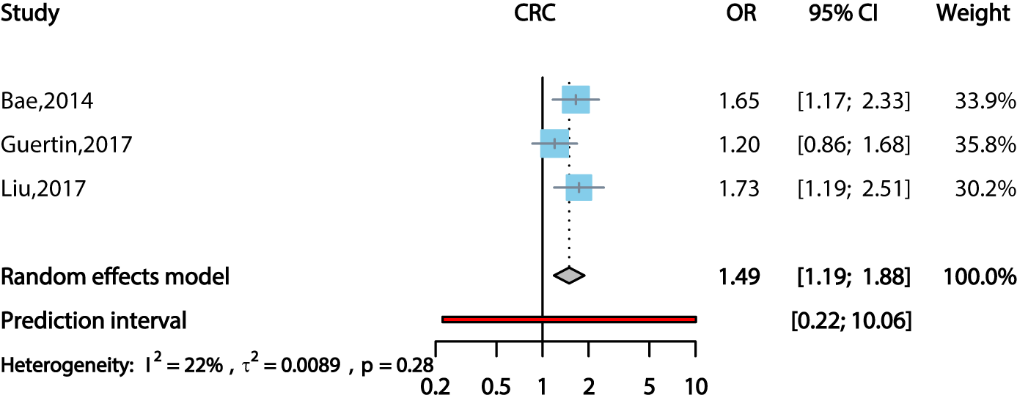


Supplementary Figure 14. Forest plot showing the risk of the effect of Trimethylamine N-oxide (TMAO) on colorectal cancer (CRC). OR: odds ratio. The diamond represents the pooled risk estimate. Interstudy heterogeneity was tested using the Cochran Q statistic(t^2^) at a significance level of *P*<0.10 and quantified by the *I^2^* statistic. An *I*^2^ value ≥50% is considered to indicate substantial heterogeneity. All results are presented as odds ratio with 95% confidence intervals, using the Mantel-Haenszel method with a random-effects model.
