# Supplementary material for: Gut microbiota–derived metabolite trimethylamine-N-oxide and multiple health outcomes: an umbrella review and updated meta-analysis
Source: Am J Clin Nutr. 2022 Mar 28;116(1):230–43. doi: 10.1093/ajcn/nqac074 (PMC9257469; doi:10.1093/ajcn/nqac074)
Supplement: nqac074_Supplemental_File [file nqac074_supplemental_file.zip › Supplementary Figure1-2.docx]

**On-line Supplementary Material**

Gut microbiota-derived metabolite Trimethylamine-N-oxide (TMAO) and multiple health outcomes: an umbrella review and updated meta-analysis

Li et al.

Supplementary **Figures 1-2:**

Supplementary Figure 1. Forest plot showing the risk of the effect of Trimethylamine N-oxide (TMAO) on all-cause mortality.

Supplementary Figure 2. Forest plot showing the risk of the effect of Trimethylamine N-oxide (TMAO) on all-cause mortality by disease status.


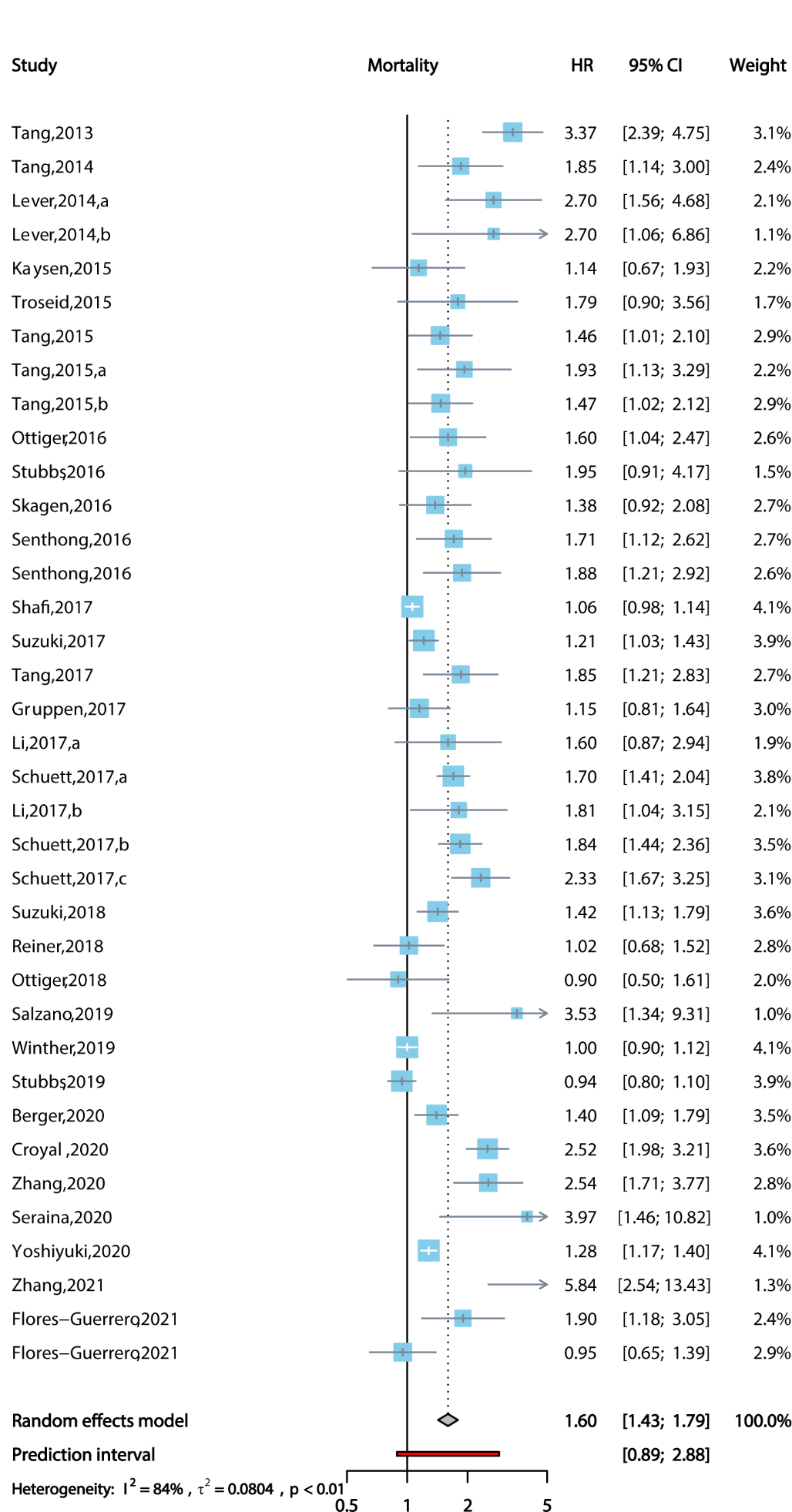


Supplementary Figure 1. Forest plot showing the risk of the effect of Trimethylamine N-oxide (TMAO) on all-cause mortality. HR: hazard ratio. The diamond represents the pooled risk estimate. Interstudy heterogeneity was tested using the Cochran Q statistic(t^2^) at a significance level of *P*<0.10 and quantified by the *I^2^* statistic. An *I*^2^ value ≥50% is considered to indicate substantial heterogeneity. All results are presented as hazard ratio with 95% confidence intervals, using the Mantel-Haenszel method with a random-effects model.


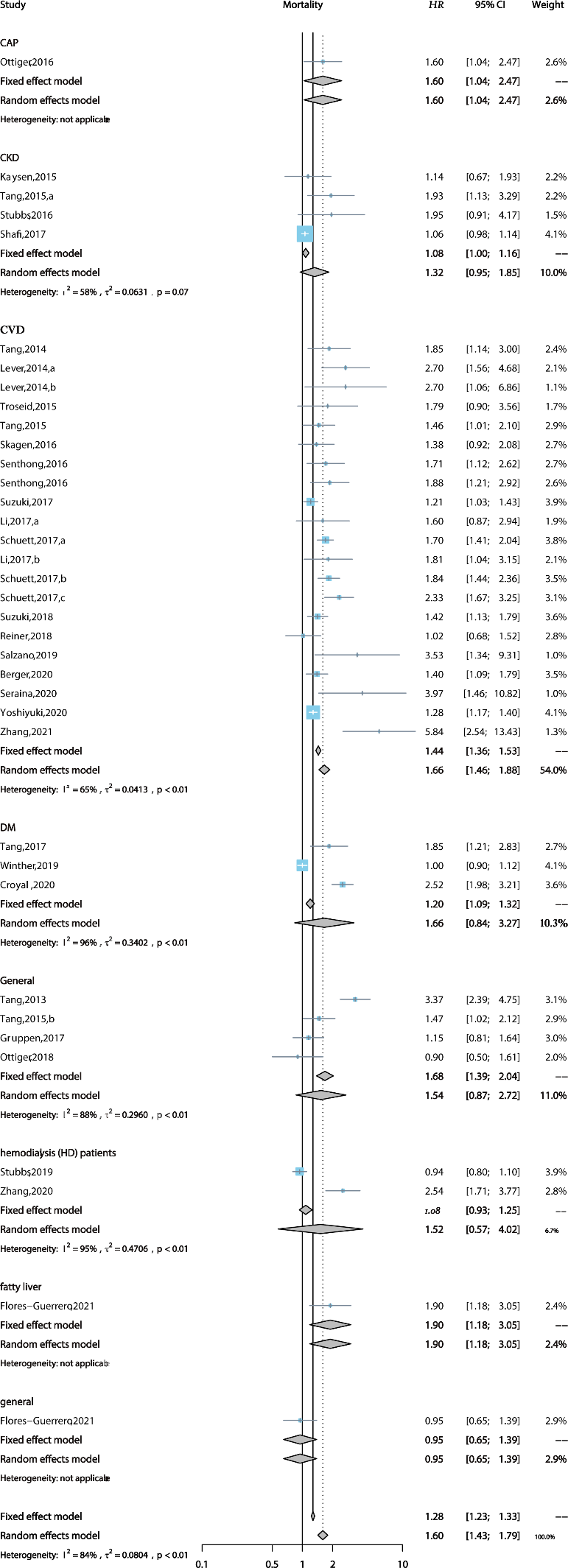


Supplementary Figure 2. Forest plot showing the risk of the effect of Trimethylamine N-oxide (TMAO) on all-cause mortality by disease status. HR: hazard ratio. The diamond represents the pooled risk estimate. Interstudy heterogeneity was tested using the Cochran Q statistic(t^2^) at a significance level of *P*<0.10 and quantified by the *I^2^* statistic. An *I*^2^ value ≥50% is considered to indicate substantial heterogeneity. All results are presented as hazard ratio with 95% confidence intervals, using the Mantel-Haenszel method with a random-effects model.
