# Supplementary material for: Gut microbiota–derived metabolite trimethylamine-N-oxide and multiple health outcomes: an umbrella review and updated meta-analysis
Source: Am J Clin Nutr. 2022 Mar 28;116(1):230–43. doi: 10.1093/ajcn/nqac074 (PMC9257469; doi:10.1093/ajcn/nqac074)
Supplement: nqac074_Supplemental_File [file nqac074_supplemental_file.zip › Supplementary Figures 3-8.docx]

**On-line Supplementary Material**

Gut microbiota-derived metabolite Trimethylamine-N-oxide (TMAO) and multiple health outcomes: an umbrella review and updated meta-analysis

Li et al.

Supplementary **Figures 3-8:**

Supplementary Figure 3. Forest plot showing the risk of the effect of Trimethylamine N-oxide (TMAO) on all-cause mortality(only included cohort studies that also adjusted for renal function).

Supplementary Figure 4. Forest plot showing the risk of the effect of Trimethylamine N-oxide (TMAO) on major adverse cardiovascular events (MACE).

Supplementary Figure 5 Forest plot showing the risk of the effect of Trimethylamine N-oxide (TMAO) on major adverse cardiovascular events (MACE) (only included cohort studies that also adjusted for renal function).

Supplementary Figure 6 Forest plot showing the risk of the effect of Trimethylamine N-oxide (TMAO) on hypertension.

Supplementary Figure 7. Forest plot showing the risk of the effect of Trimethylamine N-oxide (TMAO) on hypertension(only included cohort studies that also adjusted for renal function). RR: risk ratio.

Supplementary Figure 8. Forest plot showing the risk of the effect of Trimethylamine N-oxide (TMAO) on cardiovascular disease (CVD).


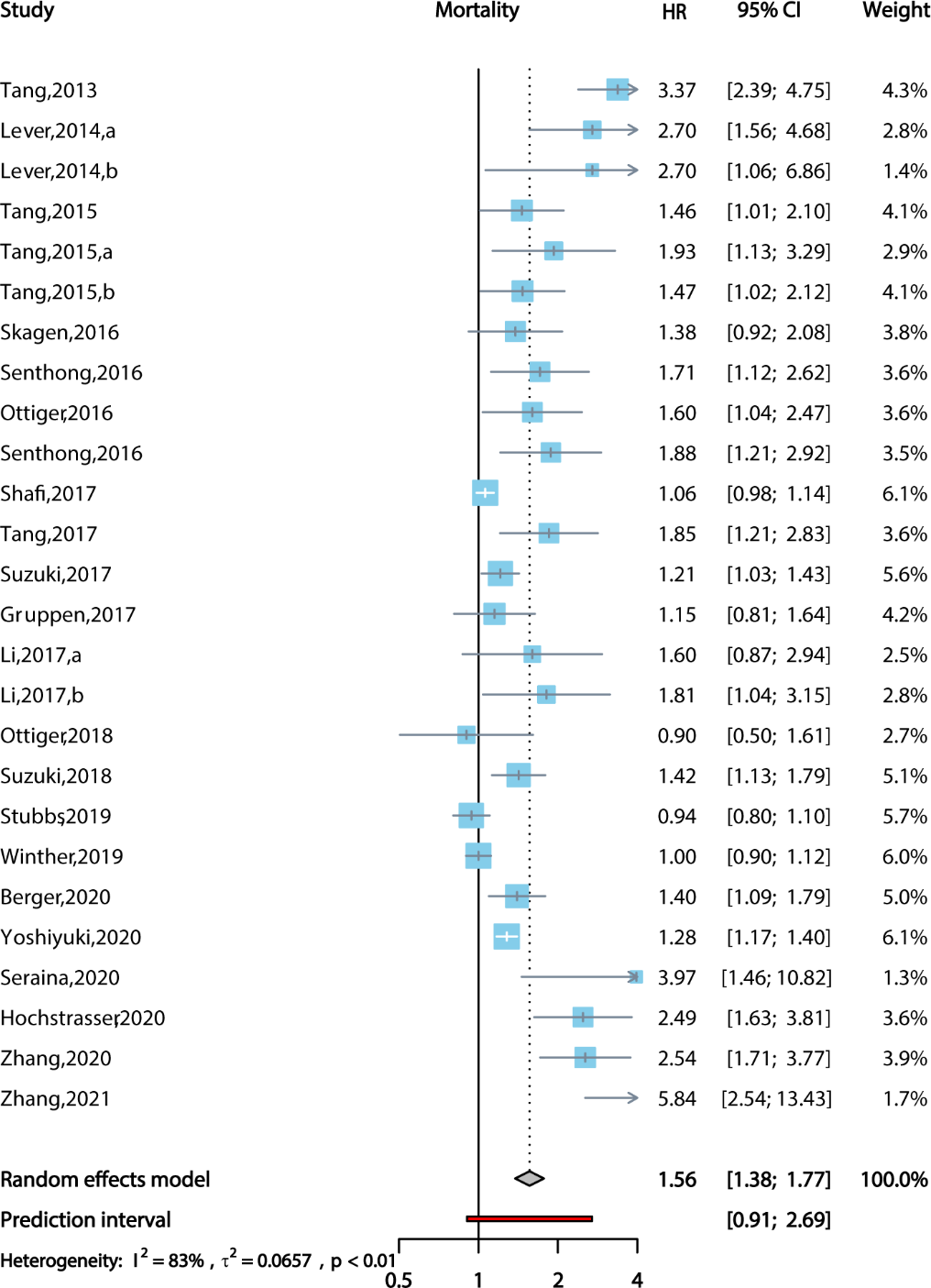


Supplementary Figure 3. Forest plot showing the risk of the effect of Trimethylamine N-oxide (TMAO) on all-cause mortality(only included cohort studies that also adjusted for renal function). HR: hazard ratio. HR: hazard ratio. The diamond represents the pooled risk estimate. Interstudy heterogeneity was tested using the Cochran Q statistic(t^2^) at a significance level of *P*<0.10 and quantified by the *I^2^* statistic. An *I*^2^ value ≥50% is considered to indicate substantial heterogeneity. All results are presented as hazard ratio with 95% confidence intervals, using the Mantel-Haenszel method with a random-effects model.


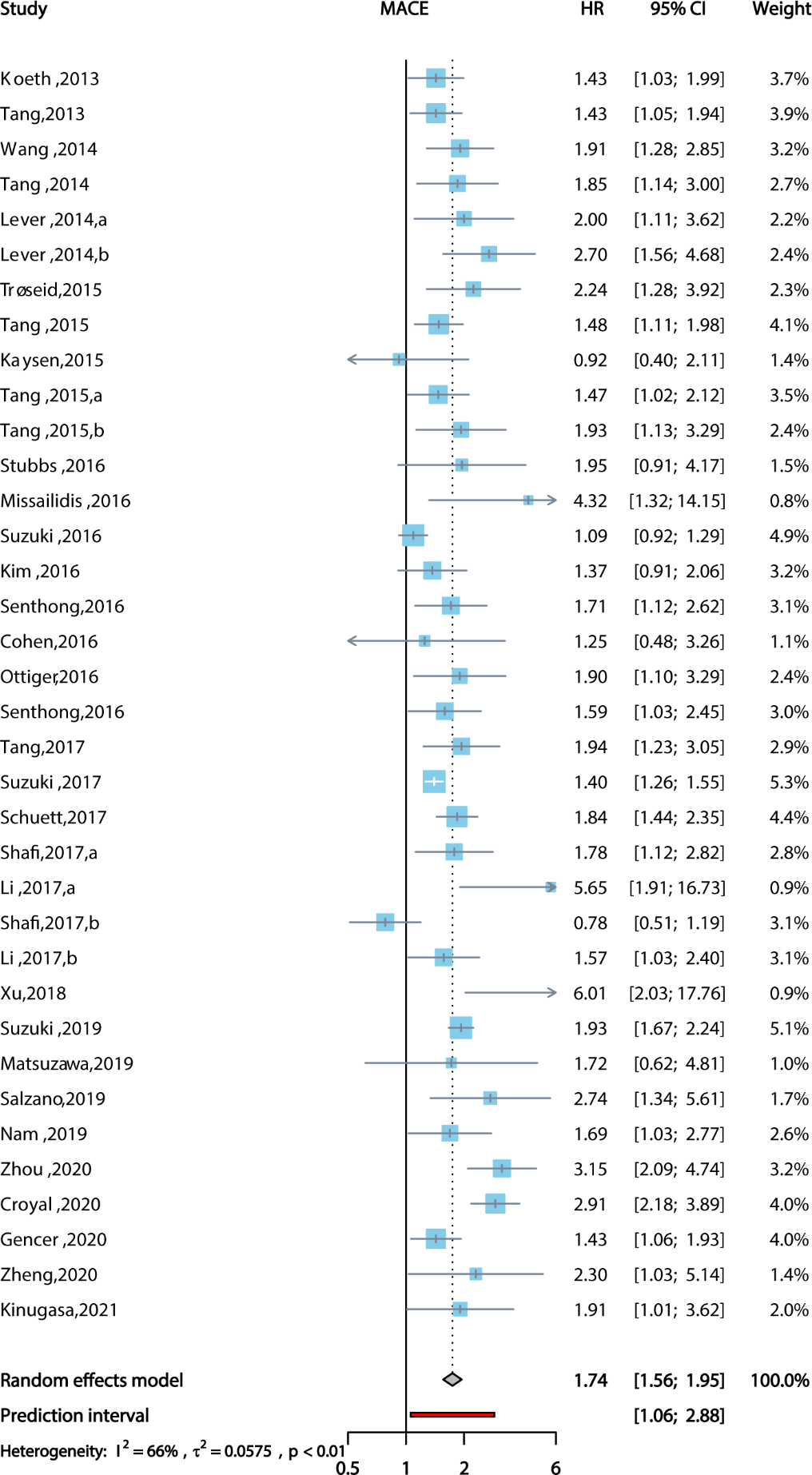


Supplementary Figure 4. Forest plot showing the risk of the effect of Trimethylamine N-oxide (TMAO) on major adverse cardiovascular events (MACE). HR: hazard ratio. The diamond represents the pooled risk estimate. Interstudy heterogeneity was tested using the Cochran Q statistic(t^2^) at a significance level of *P*<0.10 and quantified by the *I^2^* statistic. An *I*^2^ value ≥50% is considered to indicate substantial heterogeneity. All results are presented as hazard ratio with 95% confidence intervals, using the Mantel-Haenszel method with a random-effects model.


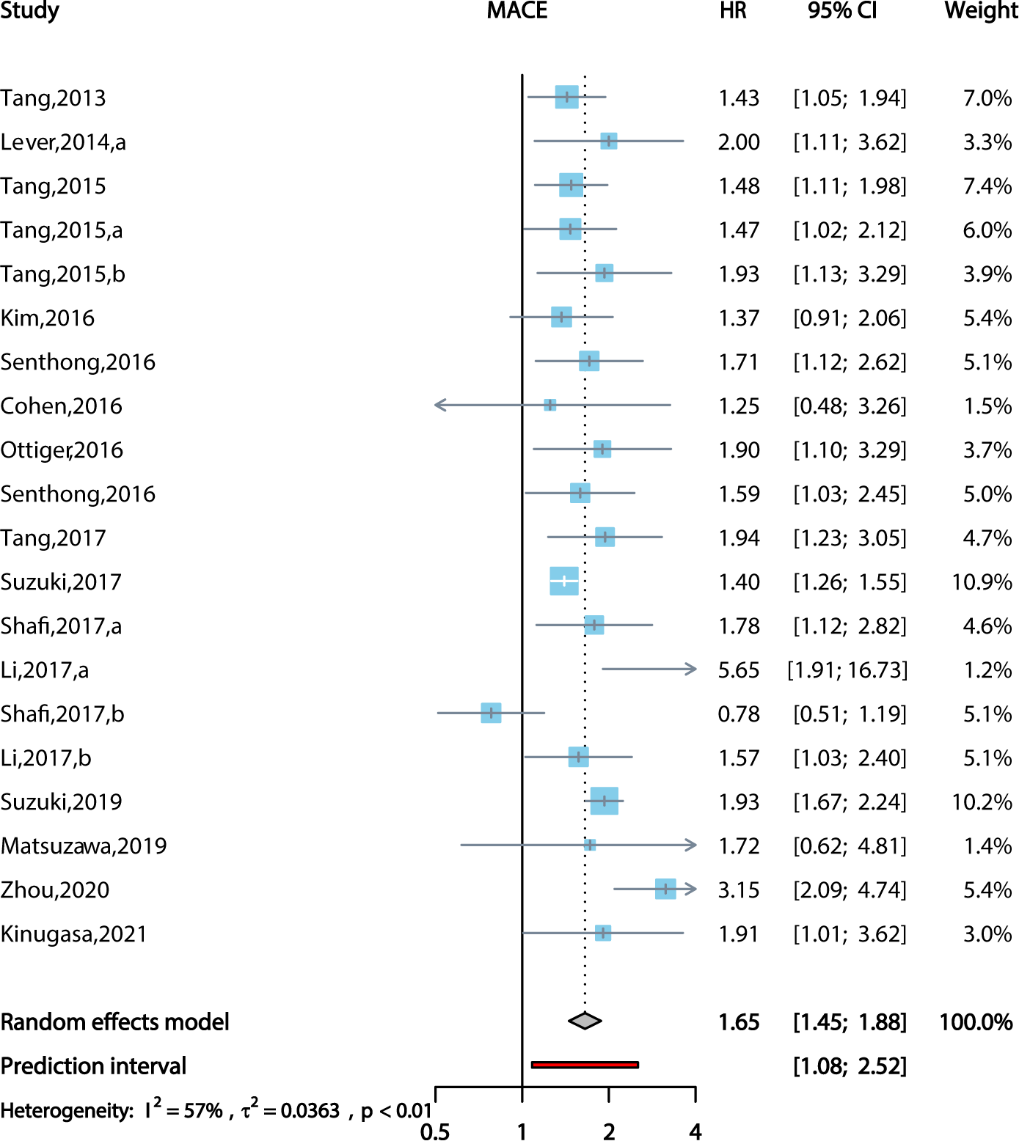


Supplementary Figure 5 Forest plot showing the risk of the effect of Trimethylamine N-oxide (TMAO) on major adverse cardiovascular events (MACE) (only included cohort studies that also adjusted for renal function). HR: hazard ratio. The diamond represents the pooled risk estimate. Interstudy heterogeneity was tested using the Cochran Q statistic(t^2^) at a significance level of *P*<0.10 and quantified by the *I^2^* statistic. An *I*^2^ value ≥50% is considered to indicate substantial heterogeneity. All results are presented as hazard ratio with 95% confidence intervals, using the Mantel-Haenszel method with a random-effects model.


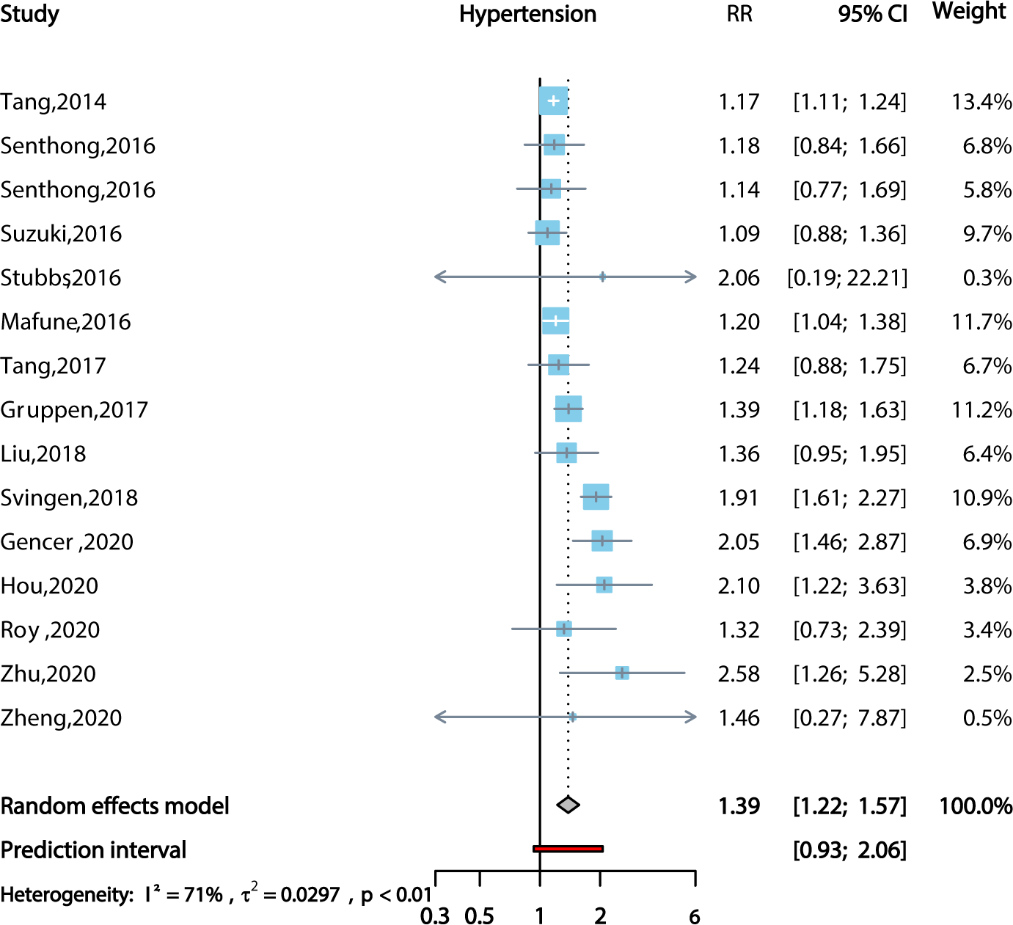


Supplementary Figure 6. Forest plot showing the risk of the effect of Trimethylamine N-oxide (TMAO) on hypertension. RR: risk ratio. The diamond represents the pooled risk estimate. Interstudy heterogeneity was tested using the Cochran Q statistic(t^2^) at a significance level of *P*<0.10 and quantified by the *I^2^* statistic. An *I*^2^ value ≥50% is considered to indicate substantial heterogeneity. All results are presented as risk ratio with 95% confidence intervals, using the Mantel-Haenszel method with a random-effects model.


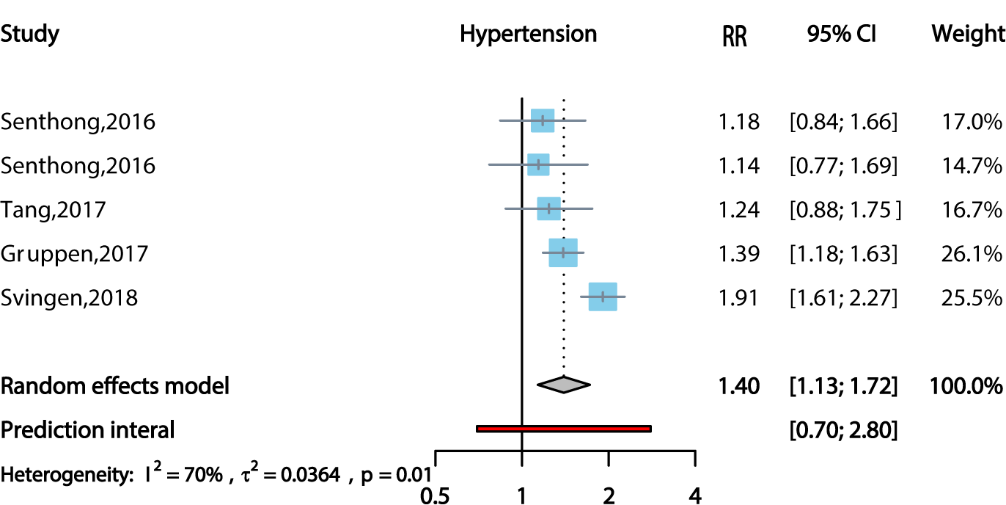


Supplementary Figure 7. Forest plot showing the risk of the effect of Trimethylamine N-oxide (TMAO) on hypertension(only included cohort studies that also adjusted for renal function). RR: risk ratio. The diamond represents the pooled risk estimate. Interstudy heterogeneity was tested using the Cochran Q statistic(t^2^) at a significance level of *P*<0.10 and quantified by the *I^2^* statistic. An *I*^2^ value ≥50% is considered to indicate substantial heterogeneity. All results are presented as risk ratio with 95% confidence intervals, using the Mantel-Haenszel method with a random-effects model.


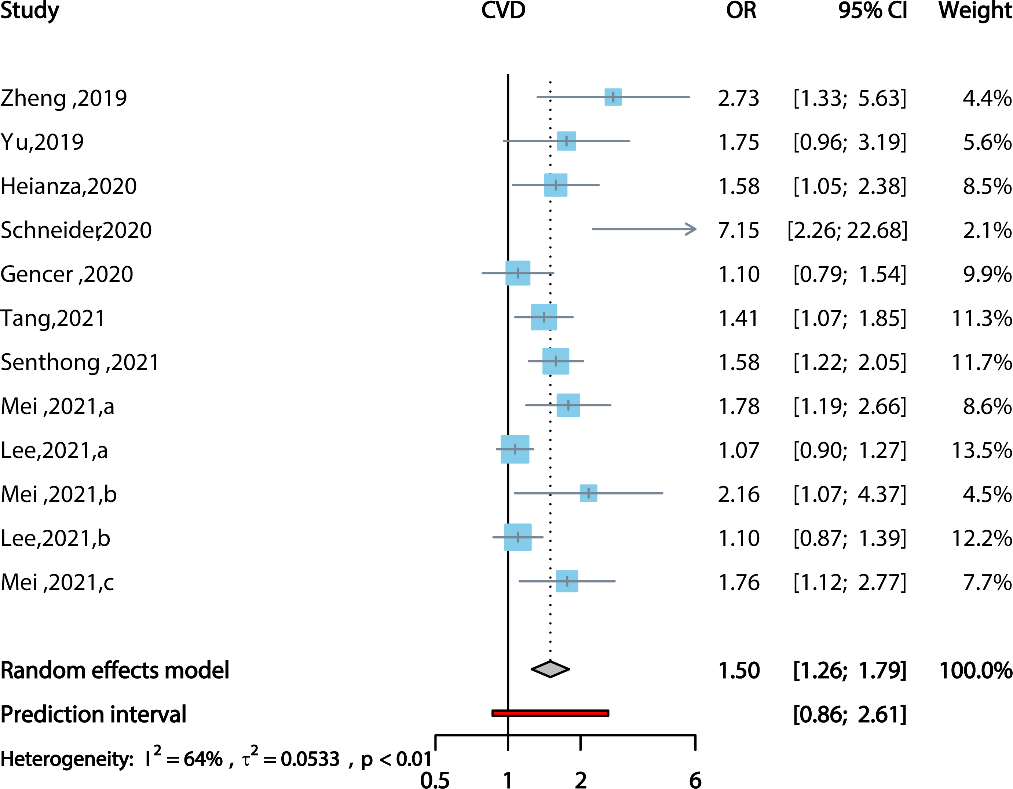


Supplementary Figure 8. Forest plot showing the risk of the effect of Trimethylamine N-oxide (TMAO) on cardiovascular disease (CVD). OR: odds ratio. The diamond represents the pooled risk estimate. Interstudy heterogeneity was tested using the Cochran Q statistic(t^2^) at a significance level of *P*<0.10 and quantified by the *I^2^* statistic. An *I*^2^ value ≥50% is considered to indicate substantial heterogeneity. All results are presented as odds ratio with 95% confidence intervals, using the Mantel-Haenszel method with a random-effects model.
