# Supplementary material for: Gut microbiota–derived metabolite trimethylamine-N-oxide and multiple health outcomes: an umbrella review and updated meta-analysis
Source: Am J Clin Nutr. 2022 Mar 28;116(1):230–43. doi: 10.1093/ajcn/nqac074 (PMC9257469; doi:10.1093/ajcn/nqac074)
Supplement: nqac074_Supplemental_File [file nqac074_supplemental_file.zip › Supplementary table 1224-Xue.docx]

**On-line Supplementary Material**

Gut microbiota-derived metabolite Trimethylamine-N-oxide (TMAO) and multiple health outcomes: an umbrella review and updated meta-analysis

Li et al.

**Supplementary Tables 1-6:**

**Supplementary Table 1.** **Criteria for quality of evidence classification in observational studies.^1-3^**

| Category | Criteria |
| --- | --- |
| Convincing, classⅠ | - No. of cases >1000 or (more than 20000 participants for continuous outcomes) - *P* < 1×10^-6^ - *I*^2^ <50% - 95% prediction intervals excluding the null value - No small study effects - No excess significance bias |
| Highly suggestive, class II | - No. of cases >1000 or (more than 20000 participants for continuous outcomes) - *P* < 1×10^-6^ - The largest component study reporting a statistically significant result |
| Suggestive, class III | - No. of cases >1000 or (more than 20000 participants for continuous outcomes) - *P* < 1×10^-3^ |
| Weak, class IV | - *P* < 0.05 |
| Not significant | - *P* > 0.05 |

**Supplementary Table 2 Description of 24 meta-analyses of TMAO levels and health outcomes included in study.**

| **Study, Year** | **Outcome** | **Disease status** | **Study design included in MA** | **Comparison** | **No of studies** | **No of cases** | **No of participants** | **Metric** | **Random effects**  **RR/OR/HR/WMD (95% CI)** | **P value** | **95% prediction interval** | **Egger’s P** | **I^2(%)** |
| --- | --- | --- | --- | --- | --- | --- | --- | --- | --- | --- | --- | --- | --- |
| **Cardiovascular outcomes** | | | | | | | | | | | | | |
| Schiattarella,2017^4^ | MACE | CKD/ non-CKD | CO | high vs low | 5 | >3363 | 15670 | HR | 1.68(1.33, 2.11) | 1.08E-05 | 1.09, 2.59 | 0 | 46 |
| Yao，2020^5^ | MACE | CHD/ ACS | CO | high vs low | 7 | >1793 | 10321 | HR | 1.58(1.35, 1.84) | 9.03E-09 | 1.15, 2.15 | 0 | 58 |
| Guasti，2021^6^ | MACE | CVD | CO | high vs low | 3 | 923 | 6220 | RR | 2.05(1.62, 2.59) | 1.71E-09 | 1.42, 2.96 | 0.62 | 48 |
| Heianza,2017^7^ | MACE | CVD/ kidney disease// DM | CO | high vs low | 19 | 3315 | 19256 | RR | 1.63(1.43, 1.85) | 1.09E-13 | 1.22, 2.17 | 0.45 | 23 |
| [Li,2020](https://pubmed.ncbi.nlm.nih.gov/?size=200&term=Li+W&cauthor_id=32959366)^8^ | MACE | CVD | CO | high vs low | 7 | 2633 | 6801 | HR | 1.68(1.44,1.96) | 4.32E-11 | 1.44, 1.96 | 0.7 | 0 |
| Farhangi,2020^9^ | stroke | CVD/ stroke/ T1DM/ T2DM | CO/CC/CS | high vs low | 5 | 1051 | 4910 | OR | 1.68(0.87, 3.24) | 0.126 | 0.38, 7.39 | 0.99 | 85 |
| Ge，2020^10^ | hypertension | healthy/ hypertensive cases | CO/CS | high vs low | 8 | 6185 | 11750 | RR | 1.12(1.06, 1.17) | 5.44E-06 | 1.00, 1.24 | 0.33 | 60 |
| Qi ，2018^11^ | CVD | CKD/ CAD/ HF | CO | high vs low | 5 | 1691 | 8139 | HR | 1.23(1.07, 1.42) | 0.005 | 0.98, 1.54 | 0.42 | 31 |
| Yang,2021^12^ | CVD | AF/HF | CO/CS/CC | high vs low | 6 | 1668 | 8837 | OR | 1.38(1.19,1.60) | 1.86E-05 | 1.01, 1.89 | 0.36 | 20 |
| **All-cause mortality** | | | | | | | | | | | | | |
| Farhangi,2020^13^ | all-cause mortality | general | CO | high vs low | 20 | >4820 | 31230 | RR | 1.47(1.29, 1.66) | 3.60E-09 | 0.87, 2.47 | 0 | 82 |
| Guasti ，2021^6^ | all-cause mortality | CVD | CO | high vs low | 3 | 179 | 3292 | HR | 3.41(2.26, 5.14) | 5.28E-09 | 2.26, 5.14 | 0.85 | 0 |
| Qi ，2018^11^ | all-cause mortality | CKD/ CAD/ HF | CO | high vs low | 11 | >815 | 7716 | HR | 1.55(1.19, 2.02) | 0.001 | 0.70, 3.40 | 0.49 | 81 |
| Heianza,2017^7^ | all-cause mortality | CVD/ kidney disease/DM | CO | high vs low | 15 | 2498 | 11676 | RR | 1.63(1.36, 1.95) | 8.08E-08 | 1.01, 2.65 | 0.01 | 46 |
| Schiattarella,2017^4^ | all-cause mortality | CKD/ non-CKD | CO | high vs low | 14 | >2849 | 15440 | HR | 1.91(1.40, 2.61) | 4.53E-05 | 0.58, 6.25 | 0.7 | 94 |
| **Diabetes mellitus** | | | | | | | | | | | | | |
| Zhuang，2019^14^ | diabetes | CVD/ diabetes/ renal disease | CO/CC | high vs low | 9 | 3318 | 12961 | OR | 1.89(1.62, 2.19) | 8.49E-17 | 1.36, 2.61 | 0.18 | 51 |
| **Blood pressure and cardiometabolic biomarkers** | | | | | | | | | | | | | |
| Farhangi,2020^15^ | DBP | T1DM/ stroke/ Healthy/ CVD | CO/CC/CS | high vs low | 7 | NA | 7813 | WMD | 0.44(-0.93, 1.81) | 0.528 | -2.85, 3.73 | 0.16 | 84 |
| Farhangi,2020^15^ | SBP | T1DM/ stroke/ Healthy/ CVD | CO/CC/CS | high vs low | 9 | NA | 15079 | WMD | 2.16(1.11, 3.21) | 5.67E-05 | -0.1, 4.42 | 0.05 | 52 |
| Dehghan,2020^16^ | BMI | general/ CVD/ T1DM/ CKD/ stroke | CO/CC/CS | high vs low | 12 | NA | 17628 | WMD | 0.56(0.03, 1.10) | 0.039 | -1.22, 2.35 | 0.49 | 97 |
| Farhangi,2020^15^ | HDL | healthy/ stroke/ CVD/ HF/ T2DM | CO/CC/CS | high vs low | 12 | NA | 21181 | WMD | -0.55(-1.78, 0.68) | 0.383 | -4.66, 3.56 | 0.1 | 94 |
| Farhangi,2020^15^ | LDL | healthy/ stroke/ CVD/ HF/ T2DM | CO/CS | high vs low | 11 | NA | 19893 | WMD | -0.52(-2.34, 1.29) | 0.572 | -4.83, 3.79 | 0.42 | 69 |
| Farhangi,2020^15^ | TC | healthy/ stroke/ CVD/ CKD | CO/CC/CS | high vs low | 7 | NA | 14251 | WMD | 0.44(-3.05, 3.92) | 0.805 | -7.62, 8.50 | 0.65 | 84 |
| Farhangi,2020^15^ | triglycerides | healthy/ stroke/ CVD/ CKD | CO/CC/CS | high vs low | 11 | NA | 15324 | WMD | 1.45(-0.33, 3.24) | 0.111 | -1.69, 4.60 | 0 | 63 |
| Farhangi,2020^17^ | CRP | healthy/ stroke/ CVD/ CKD/ T2DM | CO/CS | high vs low | 10 | NA | 13783 | WMD | 0.27(0.06, 0.48) | 0.012 | -0.27, 0.81 | 0.11 | 86 |
| **Renal Outcomes** | | | | | | | | | | | | | |
| Zeng，2021^18^ | GFR | CKD/general | CO/CC/CS | high vs low | 16 | NA | 28260 | MD | -12.86(-16.57, -9.15) | 1.11E-11 | -28.25, 2.54 | 0.89 | 98 |

^1^ NA=not available

^2^ CO= cohort study; CS= cross-sectional study; CC= case-control study.

^3^ MACE: major adverse cardiovascular events; CVD: cardiovascular disease; DBP :diastolic blood pressure; SBP: systolic blood pressure; BMI: body mass index; LDL: low - density lipoprotein cholesterol; HDL: high-density lipoprotein cholesterol; TC: total cholesterol; CRP: triglycerides and c-reactive protein; GFR: glomerular filtration rate; CKD: chronic kidney disease; CHD: coronary heart disease; ACS: acute coronary syndrome; HF: heart failure; CAD: coronary artery disease; T1DM: type 1 diabetes mellitus; T2DM: type 2 diabetes mellitus; AF: atrial fibrillation.

^4^ RR= relative risk; OR= odds ratio; HR=hazard ratio; WMD= weighed mean difference; MD= mean difference.

**Supplementary Table 3 Results of quality assessment obtained with the Newcastle-Ottawa Scale^19^ for cohort studies.**

| Author name | Study design | Selection | | | | Comparability | Outcome | | | Final score |
| --- | --- | --- | --- | --- | --- | --- | --- | --- | --- | --- |
|  |  | Representativeness of the exposed cohort | Selection of the non-exposed cohort | Ascertainment of exposure | Demonstration that outcome of interest was not present at start of study |  | Assessment of outcome | Was follow-up long enough for outcomes to occur | Adequacy of follow up of cohorts |  |
| Koeth(2013)^20^ | CO | 0 | 0 | 1 | 1 | 2 | 1 | 1 | 1 | 7 |
| Tang(2013)^21^ | CO | 0 | 0 | 1 | 1 | 2 | 1 | 1 | 1 | 7 |
| Wang(2014)^22^ | CO | 0 | 0 | 1 | 1 | 2 | 1 | 1 | 1 | 7 |
| Troseid(2015)^23^ | CO | 0 | 0 | 1 | 1 | 2 | 1 | 1 | 1 | 7 |
| Tang(2014)^24^ | CO | 0 | 0 | 1 | 1 | 1 | 1 | 1 | 1 | 6 |
| Lever(2014)^25^ | CO | 0 | 0 | 1 | 1 | 2 | 1 | 1 | 1 | 7 |
| Tang(2015)^26^ | CO | 0 | 0 | 1 | 1 | 2 | 1 | 1 | 1 | 7 |
| Stubbs(2016)^27^ | CO | 0 | 0 | 1 | 1 | 1 | 1 | 1 | 1 | 6 |
| Missailidis(2016)^28^ | CO | 0 | 0 | 1 | 1 | 1 | 1 | 1 | 1 | 6 |
| Skagen(2016)^29^ | CO | 0 | 0 | 1 | 1 | 2 | 1 | 1 | 1 | 7 |
| Suzuki(2016)^30^ | CO | 0 | 0 | 1 | 1 | 2 | 1 | 1 | 1 | 7 |
| Kim(2016)^31^ | CO | 0 | 0 | 1 | 1 | 2 | 1 | 1 | 1 | 7 |
| Senthong(2016)^32^ | CO | 0 | 0 | 1 | 1 | 2 | 1 | 1 | 1 | 7 |
| Shafi(2017)^33^ | CO | 0 | 0 | 1 | 1 | 1 | 1 | 1 | 1 | 6 |
| Robinson(2016)^34^ | CO | 0 | 0 | 1 | 1 | 1 | 1 | 1 | 1 | 6 |
| Ottiger(2016)^35^ | CO | 0 | 0 | 1 | 1 | 2 | 1 | 1 | 1 | 7 |
| Senthong(2016)^36^ | CO | 0 | 0 | 1 | 1 | 1 | 1 | 1 | 1 | 6 |
| Meyer(2016)^37^ | CO | 0 | 0 | 1 | 1 | 1 | 1 | 1 | 1 | 6 |
| Tang(2017)^38^ | CO | 0 | 0 | 1 | 1 | 2 | 1 | 1 | 1 | 7 |
| Suzuki(2017)^39^ | CO | 0 | 0 | 1 | 1 | 1 | 1 | 1 | 1 | 6 |
| Li(2017)^40^ | CO | 0 | 0 | 1 | 1 | 2 | 1 | 1 | 1 | 7 |
| Gruppen(2017)^41^ | CO | 1 | 1 | 1 | 1 | 1 | 1 | 1 | 1 | 8 |
| Svingen(2018)^42^ | CO | 0 | 0 | 1 | 1 | 1 | 1 | 1 | 1 | 6 |
| Haghikia(2018)^43^ | CO | 0 | 0 | 1 | 1 | 2 | 1 | 1 | 1 | 7 |
| Suzuki(2019)^44^ | CO | 0 | 0 | 1 | 1 | 1 | 1 | 1 | 1 | 6 |
| Reiner(2019)^45^ | CO | 0 | 0 | 1 | 1 | 2 | 1 | 1 | 1 | 7 |
| Stubbs(2019)^46^ | CO | 0 | 0 | 1 | 1 | 1 | 1 | 1 | 1 | 6 |
| Winter(2019)^47^ | CO | 0 | 0 | 1 | 1 | 2 | 1 | 1 | 1 | 7 |
| Salzano(2020)^48^ | CO | 0 | 0 | 1 | 1 | 1 | 1 | 1 | 1 | 6 |
| Zhou(2020)^49^ | CO | 0 | 0 | 1 | 1 | 1 | 1 | 1 | 1 | 6 |
| Tang(2015)^50^ | CO | 0 | 0 | 1 | 1 | 1 | 1 | 1 | 1 | 6 |
| Kaysen(2015)^51^ | CO | 0 | 0 | 1 | 1 | 2 | 1 | 1 | 1 | 7 |
| Schuett(2017)^52^ | CO | 0 | 0 | 1 | 1 | 1 | 1 | 1 | 1 | 6 |
| Zhu(2020)^53^ | CO | 0 | 0 | 1 | 1 | 1 | 1 | 1 | 1 | 6 |
| Nam(2019)^54^ | CO | 0 | 0 | 1 | 1 | 2 | 1 | 1 | 0 | 6 |
| Berger(2020)^55^ | CO | 0 | 0 | 1 | 1 | 2 | 1 | 1 | 1 | 7 |
| Croyal(2020)^56^ | CO | 0 | 0 | 1 | 1 | 1 | 1 | 1 | 1 | 6 |
| Zhang(2020)^57^ | CO | 0 | 0 | 1 | 1 | 2 | 1 | 1 | 1 | 7 |
| Guerrero(2021)^58^ | CO | 0 | 0 | 1 | 1 | 2 | 1 | 1 | 1 | 7 |
| Fu(2021)^59^ | CO | 0 | 0 | 1 | 1 | 1 | 1 | 1 | 1 | 6 |
| Zhang(2021)^60^ | CO | 0 | 0 | 1 | 1 | 2 | 1 | 1 | 1 | 7 |
| Winter(2021)^61^ | CO | 0 | 0 | 1 | 1 | 2 | 1 | 1 | 1 | 7 |
| Xu(2017)^62^ | CO | 0 | 1 | 1 | 1 | 1 | 1 | 1 | 1 | 7 |
| Guerrero(2021)^63^ | CO | 0 | 0 | 1 | 1 | 1 | 1 | 1 | 1 | 6 |
| Seraina(2020)^64^ | CO | 0 | 0 | 1 | 1 | 1 | 1 | 1 | 1 | 6 |
| Guerrero(2021)^65^ | CO | 0 | 1 | 1 | 1 | 1 | 1 | 1 | 1 | 7 |
| [Yoshiyuki](https://pubmed.ncbi.nlm.nih.gov/?size=200&term=Yazaki+Y&cauthor_id=32598563)(2020)^66^ | CO | 0 | 1 | 1 | 1 | 1 | 1 | 1 | 1 | 7 |
| Ottiger(2018)^67^ | CO | 0 | 1 | 1 | 1 | 1 | 1 | 1 | 1 | 7 |
| Lee(2021)^68^ | CO | 0 | 1 | 1 | 1 | 2 | 1 | 1 | 1 | 8 |
| Lemaitre(2021)^69^ | CO | 0 | 1 | 1 | 1 | 2 | 1 | 1 | 1 | 8 |
| Senthong(2021)^70^ | CO | 0 | 1 | 1 | 1 | 1 | 1 | 1 | 1 | 7 |
| Kinugasa(2021)^71^ | CO | 0 | 0 | 1 | 1 | 1 | 1 | 1 | 1 | 6 |

^1^ CO= cohort study

^2^ Final score ≥6 were deemed to be high quality^10^

**Supplementary Table 4 Results of quality assessment obtained with the Newcastle-Ottawa Scale^19^ for case-control studies.**

| Author name | Study design | Selection | | | | Comparability | Outcome | | | Final score |
| --- | --- | --- | --- | --- | --- | --- | --- | --- | --- | --- |
|  |  | Is the case definition adequate? | Representativeness of the cases | Selection of Controls | Definition of Controls |  | Ascertainment of exposure | Same method of ascertainment for cases and controls | Non-Response rate |  |
| Ferré(2017)^72^ | CC | 1 | 0 | 0 | 1 | 2 | 1 | 1 | 1 | 7 |
| Shan(2017)^73^ | CC | 1 | 0 | 0 | 1 | 2 | 1 | 1 | 1 | 7 |
| Nie(2018)^74^ | CC | 1 | 0 | 0 | 1 | 1 | 1 | 1 | 1 | 6 |
| Obeid(2016)^75^ | CC | 1 | 0 | 0 | 1 | 1 | 1 | 1 | 1 | 6 |
| Zheng(2019)^76^ | CC | 1 | 1 | 1 | 1 | 2 | 1 | 1 | 0 | 8 |
| Li(2018)^77^ | CC | 1 | 1 | 0 | 0 | 2 | 1 | 1 | 0 | 6 |
| Yu(2019)^78^ | CC | 1 | 1 | 0 | 1 | 1 | 1 | 1 | 0 | 6 |
| Huo(2019)^79^ | CC | 1 | 1 | 0 | 1 | 2 | 1 | 1 | 0 | 7 |
| Hou(2020)^80^ | CC | 1 | 1 | 0 | 0 | 1 | 1 | 1 | 0 | 5 |
| Heianza(2020)^81^ | CC | 1 | 1 | 0 | 1 | 1 | 1 | 1 | 1 | 7 |
| Schneider(2020)^82^ | CC | 1 | 1 | 0 | 0 | 2 | 1 | 1 | 1 | 7 |
| Gencer(2020)^83^ | CC | 0 | 0 | 1 | 0 | 1 | 1 | 0 | 1 | 4 |
| Sun(2021)^84^ | CC | 1 | 1 | 1 | 1 | 2 | 1 | 1 | 1 | 9 |
| Tang(2021)^85^ | CC | 1 | 1 | 1 | 1 | 2 | 1 | 1 | 0 | 8 |
| Bae(2014)^86^ | CC | 1 | 1 | 1 | 1 | 1 | 1 | 1 | 0 | 8 |
| Guertin(2017)^87^ | CC | 1 | 1 | 1 | 1 | 1 | 1 | 1 | 0 | 8 |
| Liu(2017)^88^ | CC | 1 | 1 | 1 | 1 | 1 | 1 | 1 | 0 | 8 |

^1^ CC= case-control study

^2^ Final score ≥6 were deemed to be high quality^10^

**Supplementary Table 5 Results of quality assessment obtained with the Newcastle-Ottawa Scale^19^ for cross-sectional studies.**

| Author name | Study design | Selection | | | | Comparability | Outcome | | Final score |
| --- | --- | --- | --- | --- | --- | --- | --- | --- | --- |
|  |  | Representativeness of the sample | Sample size | Non-respondents | Ascertainment of the exposure |  | Assessment of the outcome | Statistical test |  |
| Mente(2015)^89^ | CS | 1 | 1 | 0 | 2 | 2 | 2 | 1 | 9 |
| Rohrmann(2016)^90^ | CS | 1 | 1 | 0 | 2 | 1 | 2 | 1 | 8 |
| Mafune(2016)^91^ | CS | 0 | 1 | 0 | 2 | 2 | 2 | 1 | 8 |
| Randrianarisoa(2016)^92^ | CS | 1 | 1 | 0 | 2 | 1 | 2 | 1 | 8 |
| Aslibekyan(2017)^93^ | CS | 0 | 1 | 0 | 2 | 1 | 2 | 1 | 7 |
| Krüger(2017)^94^ | CS | 0 | 1 | 0 | 2 | 2 | 2 | 1 | 8 |
| Liu(2018)^95^ | CS | 0 | 0 | 0 | 2 | 2 | 2 | 1 | 7 |
| Matsuzawa(2019)^96^ | CS | 0 | 1 | 0 | 2 | 2 | 2 | 1 | 8 |
| Papandreou(2018)^97^ | CS | 1 | 1 | 0 | 2 | 2 | 2 | 1 | 9 |
| Wu(2020)^98^ | CS | 1 | 1 | 0 | 2 | 1 | 2 | 1 | 8 |
| Roy(2020)^99^ | CS | 1 | 1 | 0 | 2 | 2 | 2 | 1 | 9 |
| Zheng(2020)^100^ | CS | 0 | 1 | 0 | 2 | 2 | 2 | 1 | 8 |
| Mei(2021)^101^ | CS | 1 | 1 | 0 | 2 | 2 | 2 | 1 | 9 |

^1^ CS= cross-sectional study

^2^ Final score ≥6 were deemed to be high quality^10^

**Supplementary Table 6 Information of other outcomes included in the review based on single studies.**

| **Author** | **Year** | **Study design** | **Disease status** | **Comparison/Unit** | **Outcome** | **Number of Cases** | **Total Participants** | **Type of metrix** | **Relative risk** | **Lower 95% CI** | **Higher 95% CI** | **P value** |
| --- | --- | --- | --- | --- | --- | --- | --- | --- | --- | --- | --- | --- |
| Huang^102^ | 2020 | CC | PE/healthy | high vs low | PE | 66 | 264 | OR | 1 | 0.47 | 2.14 | 0.867 |
| Barrea^103^ | 2018 | CS | general | high vs low | Mets | -- | 137 | OR | 2.36 | 1.73 | 3.23 | <0.001 |
| Liu^104^ | 2021 | CS | T2DM | high vs low | DR | 72 | 122 | OR | 7.42 | 1.72 | 32 | 0.002 |
| Liu^105^ | 2020 | CC | Hip fracture/healthy | high vs low | hip fracture | 286 | 572 | OR | 3.68 | 2.04 | 5.96 | <0.001 |
| Chung^106^ | 2021 | CO | PD/healthy | high vs low | PD | 85 | 105 | HR | 7.565 | 1.00 | 57.02 | 0.050 |
| Mondul^107^ | 2015 | CC | prostate cancer/healthy | high vs low | prostate cancer | 200 | 400 | OR | 1.36 | 1.02 | 1.81 | 0.039 |
| Liu^108^ | 2018 | CC | PLC/healthy | high vs low | PLC | 671 | 1342 | OR | 2.85 | 1.59 | 5.11 | 0.005 |
| Huang^109^ | 2020 | CC | pancreatic cancer/healthy | high vs low | pancreatic cancer | 187 | 549 | OR | 2.36 | 1.3 | 4.26 | 0.02 |
| Chen^110^ | 2016 | CS | general | high vs low | NAFLD | 914 | 1628 | OR | 1.53 | 1.08 | 2.17 | 0.023 |
| Leon^111^ | 2021 | CS | obesity | high vs low | NAFLD | 319 | 357 | OR | 2.77 | 1.38 | 5.55 | 0.004 |

^1^ PE: preeclampsia; Mets: metabolic syndrome; DR: diabetic retinopathy; PD: Parkinson’s disease; PLC: primary liver cancer; NAFLD: non-alcoholic fatty liver disease.

^2^ CC: case-control study; CO: cohort study; CS: cross-sectional study.

^3^ OR: odds ratio; HR: hazard ratio.

**References**

1. Li X, Meng X, Timofeeva M, Tzoulaki I, Tsilidis KK, Ioannidis JP, et al. Serum uric acid levels and multiple health outcomes: umbrella review of evidence from observational studies, randomised controlled trials, and Mendelian randomisation studies. BMJ. 2017; 357:j2376.

2. Kalliala I, Markozannes G, Gunter MJ, Paraskevaidis E, Gabra H, Mitra A, et al. Obesity and gynaecological and obstetric conditions: umbrella review of the literature. BMJ. 2017; 359:j4511.

3. Kyrgiou M, Kalliala I, Markozannes G, Gunter MJ, Paraskevaidis E, Gabra H, et al. Adiposity and cancer at major anatomical sites: umbrella review of the literature. BMJ. 2017; 356:j477.

4. Schiattarella GG, Sannino A, Toscano E, Giugliano G, Gargiulo G, Franzone A, et al. Gut microbe-generated metabolite trimethylamine-N-oxide as cardiovascular risk biomarker: a systematic review and dose-response meta-analysis. Eur Heart J. 2017; 38:2948-56.

5. Yao ME, Liao PD, Zhao XJ, Wang L. Trimethylamine-N-oxide has prognostic value in coronary heart disease: a meta-analysis and dose-response analysis. BMC Cardiovasc Disord. 2020; 20:7.

6. Guasti L, Galliazzo S, Molaro M, Visconti E, Pennella B, Gaudio GV, et al. TMAO as a biomarker of cardiovascular events: a systematic review and meta-analysis. Intern Emerg Med. 2021; 16:201-7.

7. Heianza Y, Ma W, Manson JE, Rexrode KM, Qi L. Gut Microbiota Metabolites and Risk of Major Adverse Cardiovascular Disease Events and Death: A Systematic Review and Meta-Analysis of Prospective Studies. J Am Heart Assoc. 2017; 6.

8. Li W, Huang A, Zhu H, Liu X, Huang X, Huang Y, et al. Gut microbiota-derived trimethylamine N-oxide is associated with poor prognosis in patients with heart failure. Med J Aust. 2020; 213:374-9.

9. Farhangi MA, Vajdi M, Asghari-Jafarabadi M. Gut microbiota-associated metabolite trimethylamine N-Oxide and the risk of stroke: a systematic review and dose-response meta-analysis. Nutr J. 2020; 19:76.

10. Ge X, Zheng L, Zhuang R, Yu P, Xu Z, Liu G, et al. The Gut Microbial Metabolite Trimethylamine N-Oxide and Hypertension Risk: A Systematic Review and Dose-Response Meta-analysis. Adv Nutr. 2020; 11:66-76.

11. Qi J, You T, Li J, Pan T, Xiang L, Han Y, et al. Circulating trimethylamine N-oxide and the risk of cardiovascular diseases: a systematic review and meta-analysis of 11 prospective cohort studies. J Cell Mol Med. 2018; 22:185-94.

12. Yang WT, Yang R, Zhao Q, Li XD, Wang YT. A systematic review and meta-analysis of the gut microbiota-dependent metabolite trimethylamine N-oxide with the incidence of atrial fibrillation. Ann Palliat Med. 2021; 10:11512-23.

13. Farhangi MA. Gut microbiota-dependent trimethylamine N-oxide and all-cause mortality: Findings from an updated systematic review and meta-analysis. Nutrition. 2020; 78:110856.

14. Zhuang R, Ge X, Han L, Yu P, Gong X, Meng Q, et al. Gut microbe-generated metabolite trimethylamine N-oxide and the risk of diabetes: A systematic review and dose-response meta-analysis. Obes Rev. 2019; 20:883-94.

15. Abbasalizad Farhangi M, Vajdi M. Gut microbiota-associated trimethylamine N-oxide and increased cardiometabolic risk in adults: a systematic review and dose-response meta-analysis. Nutr Rev. 2021; 79:1022-42.

16. Dehghan P, Farhangi MA, Nikniaz L, Nikniaz Z, Asghari-Jafarabadi M. Gut microbiota-derived metabolite trimethylamine N-oxide (TMAO) potentially increases the risk of obesity in adults: An exploratory systematic review and dose-response meta- analysis. Obes Rev. 2020; 21:e12993.

17. Farhangi MA, Vajdi M. Novel findings of the association between gut microbiota-derived metabolite trimethylamine N-oxide and inflammation: results from a systematic review and dose-response meta-analysis. Crit Rev Food Sci Nutr. 2020; 60:2801-23.

18. Zeng Y, Guo M, Fang X, Teng F, Tan X, Li X, et al. Gut Microbiota-Derived Trimethylamine N-Oxide and Kidney Function: A Systematic Review and Meta-Analysis. Adv Nutr. 2021; 12:1286-304.

19. Zhang L, Hu P, Chen X, Bie P. Newcastle-Ottawa Scale (NOS) for assessing the quality of nonrandomized trials. 2014.

20. Koeth RA, Wang Z, Levison BS, Buffa JA, Org E, Sheehy BT, et al. Intestinal microbiota metabolism of L-carnitine, a nutrient in red meat, promotes atherosclerosis. Nat Med. 2013; 19:576-85.

21. Tang WH, Wang Z, Levison BS, Koeth RA, Britt EB, Fu X, et al. Intestinal microbial metabolism of phosphatidylcholine and cardiovascular risk. N Engl J Med. 2013; 368:1575-84.

22. Wang Z, Tang WH, Buffa JA, Fu X, Britt EB, Koeth RA, et al. Prognostic value of choline and betaine depends on intestinal microbiota-generated metabolite trimethylamine-N-oxide. Eur Heart J. 2014; 35:904-10.

23. Troseid M, Ueland T, Hov JR, Svardal A, Gregersen I, Dahl CP, et al. Microbiota-dependent metabolite trimethylamine-N-oxide is associated with disease severity and survival of patients with chronic heart failure. J Intern Med. 2015; 277:717-26.

24. Tang WH, Wang Z, Fan Y, Levison B, Hazen JE, Donahue LM, et al. Prognostic value of elevated levels of intestinal microbe-generated metabolite trimethylamine-N-oxide in patients with heart failure: refining the gut hypothesis. J Am Coll Cardiol. 2014; 64:1908-14.

25. Lever M, George PM, Slow S, Bellamy D, Young JM, Ho M, et al. Betaine and Trimethylamine-N-Oxide as Predictors of Cardiovascular Outcomes Show Different Patterns in Diabetes Mellitus: An Observational Study. PLoS One. 2014; 9:e114969.

26. Tang WH, Wang Z, Kennedy DJ, Wu Y, Buffa JA, Agatisa-Boyle B, et al. Gut microbiota-dependent trimethylamine N-oxide (TMAO) pathway contributes to both development of renal insufficiency and mortality risk in chronic kidney disease. Circ Res. 2015; 116:448-55.

27. Stubbs JR, House JA, Ocque AJ, Zhang S, Johnson C, Kimber C, et al. Serum Trimethylamine-N-Oxide is Elevated in CKD and Correlates with Coronary Atherosclerosis Burden. J Am Soc Nephrol. 2016; 27:305-13.

28. Missailidis C, Hallqvist J, Qureshi AR, Barany P, Heimburger O, Lindholm B, et al. Serum Trimethylamine-N-Oxide Is Strongly Related to Renal Function and Predicts Outcome in Chronic Kidney Disease. PLoS One. 2016; 11:e0141738.

29. Skagen K, Troseid M, Ueland T, Holm S, Abbas A, Gregersen I, et al. The Carnitine-butyrobetaine-trimethylamine-N-oxide pathway and its association with cardiovascular mortality in patients with carotid atherosclerosis. Atherosclerosis. 2016; 247:64-9.

30. Suzuki T, Heaney LM, Bhandari SS, Jones DJ, Ng LL. Trimethylamine N-oxide and prognosis in acute heart failure. Heart. 2016; 102:841-8.

31. Kim RB, Morse BL, Djurdjev O, Tang M, Muirhead N, Barrett B, et al. Advanced chronic kidney disease populations have elevated trimethylamine N-oxide levels associated with increased cardiovascular events. Kidney Int. 2016; 89:1144-52.

32. Senthong V, Wang Z, Li XS, Fan Y, Wu Y, Tang WH, et al. Intestinal Microbiota-Generated Metabolite Trimethylamine-N-Oxide and 5-Year Mortality Risk in Stable Coronary Artery Disease: The Contributory Role of Intestinal Microbiota in a COURAGE-Like Patient Cohort. J Am Heart Assoc. 2016; 5.

33. Shafi T, Powe NR, Meyer TW, Hwang S, Hai X, Melamed ML, et al. Trimethylamine N-Oxide and Cardiovascular Events in Hemodialysis Patients. J Am Soc Nephrol. 2017; 28:321-31.

34. Robinson-Cohen C, Newitt R, Shen DD, Rettie AE, Kestenbaum BR, Himmelfarb J, et al. Association of FMO3 Variants and Trimethylamine N-Oxide Concentration, Disease Progression, and Mortality in CKD Patients. PLoS One. 2016; 11:e0161074.

35. Ottiger M, Nickler M, Steuer C, Odermatt J, Huber A, Christ-Crain M, et al. Trimethylamine-N-oxide (TMAO) predicts fatal outcomes in community-acquired pneumonia patients without evident coronary artery disease. Eur J Intern Med. 2016; 36:67-73.

36. Senthong V, Wang Z, Fan Y, Wu Y, Hazen SL, Tang WH. Trimethylamine N-Oxide and Mortality Risk in Patients With Peripheral Artery Disease. J Am Heart Assoc. 2016; 5.

37. Meyer KA, Benton TZ, Bennett BJ, Jacobs DR, Jr., Lloyd-Jones DM, Gross MD, et al. Microbiota-Dependent Metabolite Trimethylamine N-Oxide and Coronary Artery Calcium in the Coronary Artery Risk Development in Young Adults Study (CARDIA). J Am Heart Assoc. 2016; 5.

38. Tang WH, Wang Z, Li XS, Fan Y, Li DS, Wu Y, et al. Increased Trimethylamine N-Oxide Portends High Mortality Risk Independent of Glycemic Control in Patients with Type 2 Diabetes Mellitus. Clin Chem. 2017; 63:297-306.

39. Suzuki T, Heaney LM, Jones DJ, Ng LL. Trimethylamine N-oxide and Risk Stratification after Acute Myocardial Infarction. Clin Chem. 2017; 63:420-8.

40. Li XS, Obeid S, Klingenberg R, Gencer B, Mach F, Raber L, et al. Gut microbiota-dependent trimethylamine N-oxide in acute coronary syndromes: a prognostic marker for incident cardiovascular events beyond traditional risk factors. Eur Heart J. 2017; 38:814-24.

41. Gruppen EG, Garcia E, Connelly MA, Jeyarajah EJ, Otvos JD, Bakker SJL, et al. TMAO is Associated with Mortality: Impact of Modestly Impaired Renal Function. Sci Rep. 2017; 7:13781.

42. Svingen GFT, Zuo H, Ueland PM, Seifert R, Loland KH, Pedersen ER, et al. Increased plasma trimethylamine-N-oxide is associated with incident atrial fibrillation. Int J Cardiol. 2018; 267:100-6.

43. Haghikia A, Li XS, Liman TG, Bledau N, Schmidt D, Zimmermann F, et al. Gut Microbiota-Dependent Trimethylamine N-Oxide Predicts Risk of Cardiovascular Events in Patients With Stroke and Is Related to Proinflammatory Monocytes. Arterioscler Thromb Vasc Biol. 2018; 38:2225-35.

44. Suzuki T, Yazaki Y, Voors AA, Jones DJL, Chan DCS, Anker SD, et al. Association with outcomes and response to treatment of trimethylamine N-oxide in heart failure: results from BIOSTAT-CHF. Eur J Heart Fail. 2019; 21:877-86.

45. Reiner MF, Muller D, Gobbato S, Stalder O, Limacher A, Bonetti NR, et al. Gut microbiota-dependent trimethylamine-N-oxide (TMAO) shows a U-shaped association with mortality but not with recurrent venous thromboembolism. Thromb Res. 2019; 174:40-7.

46. Stubbs JR, Stedman MR, Liu S, Long J, Franchetti Y, West RE, 3rd, et al. Trimethylamine N-Oxide and Cardiovascular Outcomes in Patients with ESKD Receiving Maintenance Hemodialysis. Clin J Am Soc Nephrol. 2019; 14:261-7.

47. Winther SA, Ollgaard JC, Tofte N, Tarnow L, Wang Z, Ahluwalia TS, et al. Utility of Plasma Concentration of Trimethylamine N-Oxide in Predicting Cardiovascular and Renal Complications in Individuals With Type 1 Diabetes. Diabetes Care. 2019; 42:1512-20.

48. Salzano A, Israr MZ, Yazaki Y, Heaney LM, Kanagala P, Singh A, et al. Combined use of trimethylamine N-oxide with BNP for risk stratification in heart failure with preserved ejection fraction: findings from the DIAMONDHFpEF study. Eur J Prev Cardiol. 2020; 27:2159-62.

49. Zhou X, Jin M, Liu L, Yu Z, Lu X, Zhang H. Trimethylamine N-oxide and cardiovascular outcomes in patients with chronic heart failure after myocardial infarction. ESC Heart Fail. 2020; 7:188-93.

50. Tang WH, Wang Z, Shrestha K, Borowski AG, Wu Y, Troughton RW, et al. Intestinal microbiota-dependent phosphatidylcholine metabolites, diastolic dysfunction, and adverse clinical outcomes in chronic systolic heart failure. J Card Fail. 2015; 21:91-6.

51. Kaysen GA, Johansen KL, Chertow GM, Dalrymple LS, Kornak J, Grimes B, et al. Associations of Trimethylamine N-Oxide With Nutritional and Inflammatory Biomarkers and Cardiovascular Outcomes in Patients New to Dialysis. J Ren Nutr. 2015; 25:351-6.

52. Schuett K, Kleber ME, Scharnagl H, Lorkowski S, Marz W, Niessner A, et al. Trimethylamine-N-oxide and Heart Failure With Reduced Versus Preserved Ejection Fraction. J Am Coll Cardiol. 2017; 70:3202-4.

53. Zhu C, Li G, Lv Z, Li J, Wang X, Kang J, et al. Association of plasma trimethylamine-N-oxide levels with post-stroke cognitive impairment: a 1-year longitudinal study. Neurol Sci. 2020; 41:57-63.

54. Nam HS, Ha J, Ji D, Kwon I, Lee HS, Han M, et al. Elevation of the Gut Microbiota Metabolite Trimethylamine N-Oxide Predicts Stroke Outcome. J Stroke. 2019; 21:350-2.

55. Berger M, Kleber ME, Delgado GE, Marz W, Andreas M, Hellstern P, et al. Trimethylamine N-Oxide and Adenosine Diphosphate-Induced Platelet Reactivity Are Independent Risk Factors for Cardiovascular and All-Cause Mortality. Circ Res. 2020; 126:660-2.

56. Croyal M, Saulnier PJ, Aguesse A, Gand E, Ragot S, Roussel R, et al. Plasma Trimethylamine N-Oxide and Risk of Cardiovascular Events in Patients With Type 2 Diabetes. J Clin Endocrinol Metab. 2020; 105.

57. Zhang P, Zou JZ, Chen J, Tan X, Xiang FF, Shen B, et al. Association of trimethylamine N-Oxide with cardiovascular and all-cause mortality in hemodialysis patients. Ren Fail. 2020; 42:1004-14.

58. Flores-Guerrero JL, Oste MCJ, Baraldi PB, Connelly MA, Garcia E, Navis G, et al. Association of Circulating Trimethylamine N-Oxide and Its Dietary Determinants with the Risk of Kidney Graft Failure: Results of the TransplantLines Cohort Study. Nutrients. 2021; 13.

59. Fu D, Shen J, Li W, Wang Y, Zhong Z, Ye H, et al. Elevated Serum Trimethylamine N-Oxide Levels Are Associated with Mortality in Male Patients on Peritoneal Dialysis. Blood Purif. 2021:1-11.

60. Zhang J, Wang L, Cai J, Lei A, Liu C, Lin R, et al. Gut microbial metabolite TMAO portends prognosis in acute ischemic stroke. J Neuroimmunol. 2021; 354:577526.

61. Winther SA, Ollgaard JC, Hansen TW, von Scholten BJ, Reinhard H, Ahluwalia TS, et al. Plasma trimethylamine N-oxide and its metabolic precursors and risk of mortality, cardiovascular and renal disease in individuals with type 2-diabetes and albuminuria. PLoS One. 2021; 16:e0244402.

62. Kai-Zu XU, Lin LM, Ying WU, Jin-Hong XU, Mei-Fang WU, Cardiology DO. Relationship between plasma trimethylamine-N-oxide levels and complication risk in patients with acute myocardial infarction. Chinese Journal of Arteriosclerosis. 2017.

63. Flores-Guerrero JL, van Dijk PR, Connelly MA, Garcia E, Bilo HJG, Navis G, et al. Circulating Trimethylamine N-Oxide Is Associated with Increased Risk of Cardiovascular Mortality in Type-2 Diabetes: Results from a Dutch Diabetes Cohort (ZODIAC-59). J Clin Med. 2021; 10.

64. Hochstrasser SR, Metzger K, Vincent AM, Becker C, Keller AKJ, Beck K, et al. Trimethylamine-N-oxide (TMAO) predicts short- and long-term mortality and poor neurological outcome in out-of-hospital cardiac arrest patients. Clin Chem Lab Med. 2020; 59:393-402.

65. Flores-Guerrero JL, Post A, van Dijk PR, Connelly MA, Garcia E, Navis G, et al. Circulating trimethylamine-N-oxide is associated with all-cause mortality in subjects with nonalcoholic fatty liver disease. Liver Int. 2021; 41:2371-82.

66. Yazaki Y, Aizawa K, Israr MZ, Negishi K, Salzano A, Saitoh Y, et al. Ethnic differences in association of outcomes with trimethylamine N-oxide in acute heart failure patients. ESC Heart Fail. 2020; 7:2373-8.

67. Ottiger M, Nickler M, Steuer C, Bernasconi L, Huber A, Christ-Crain M, et al. Gut, microbiota-dependent trimethylamine-N-oxide is associated with long-term all-cause mortality in patients with exacerbated chronic obstructive pulmonary disease. Nutrition. 2018; 45:135-41 e1.

68. Lee Y, Nemet I, Wang Z, Lai HTM, de Oliveira Otto MC, Lemaitre RN, et al. Longitudinal Plasma Measures of Trimethylamine N-Oxide and Risk of Atherosclerotic Cardiovascular Disease Events in Community-Based Older Adults. J Am Heart Assoc. 2021; 10:e020646.

69. Lemaitre RN, Jensen PN, Wang Z, Fretts AM, McKnight B, Nemet I, et al. Association of Trimethylamine N-Oxide and Related Metabolites in Plasma and Incident Type 2 Diabetes: The Cardiovascular Health Study. JAMA Netw Open. 2021; 4:e2122844.

70. Senthong V, Kiatchoosakun S, Wongvipaporn C, Phetcharaburanin J, Tatsanavivat P, Sritara P, et al. Gut microbiota-generated metabolite, trimethylamine-N-oxide, and subclinical myocardial damage: a multicenter study from Thailand. Sci Rep. 2021; 11:14963.

71. Kinugasa Y, Nakamura K, Kamitani H, Hirai M, Yanagihara K, Kato M, et al. Trimethylamine N-oxide and outcomes in patients hospitalized with acute heart failure and preserved ejection fraction. ESC Heart Fail. 2021; 8:2103-10.

72. Guasch-Ferre M, Hu FB, Ruiz-Canela M, Bullo M, Toledo E, Wang DD, et al. Plasma Metabolites From Choline Pathway and Risk of Cardiovascular Disease in the PREDIMED (Prevention With Mediterranean Diet) Study. J Am Heart Assoc. 2017; 6.

73. Shan Z, Sun T, Huang H, Chen S, Chen L, Luo C, et al. Association between microbiota-dependent metabolite trimethylamine-N-oxide and type 2 diabetes. Am J Clin Nutr. 2017; 106:888-94.

74. Nie J, Xie L, Zhao BX, Li Y, Qiu B, Zhu F, et al. Serum Trimethylamine N-Oxide Concentration Is Positively Associated With First Stroke in Hypertensive Patients. Stroke. 2018; 49:2021-8.

75. Obeid R, Awwad HM, Rabagny Y, Graeber S, Herrmann W, Geisel J. Plasma trimethylamine N-oxide concentration is associated with choline, phospholipids, and methyl metabolism. Am J Clin Nutr. 2016; 103:703-11.

76. Zheng L, Zheng J, Xie Y, Li Z, Guo X, Sun G, et al. Serum gut microbe-dependent trimethylamine N-oxide improves the prediction of future cardiovascular disease in a community-based general population. Atherosclerosis. 2019; 280:126-31.

77. Li P, Zhong C, Li S, Sun T, Huang H, Chen X, et al. Plasma concentration of trimethylamine-N-oxide and risk of gestational diabetes mellitus. Am J Clin Nutr. 2018; 108:603-10.

78. Yu D, Shu XO, Rivera ES, Zhang X, Cai Q, Calcutt MW, et al. Urinary Levels of Trimethylamine-N-Oxide and Incident Coronary Heart Disease: A Prospective Investigation Among Urban Chinese Adults. J Am Heart Assoc. 2019; 8:e010606.

79. Huo X, Li J, Cao YF, Li SN, Shao P, Leng J, et al. Trimethylamine N-Oxide Metabolites in Early Pregnancy and Risk of Gestational Diabetes: A Nested Case-Control Study. J Clin Endocrinol Metab. 2019; 104:5529-39.

80. Hou L, Zhang Y, Zheng D, Shi H, Zou C, Zhang H, et al. Increasing trimethylamine N-oxide levels as a predictor of early neurological deterioration in patients with acute ischemic stroke. Neurol Res. 2020; 42:153-8.

81. Heianza Y, Ma W, DiDonato JA, Sun Q, Rimm EB, Hu FB, et al. Long-Term Changes in Gut Microbial Metabolite Trimethylamine N-Oxide and Coronary Heart Disease Risk. J Am Coll Cardiol. 2020; 75:763-72.

82. Schneider C, Okun JG, Schwarz KV, Hauke J, Zorn M, Nurnberg C, et al. Trimethylamine-N-oxide is elevated in the acute phase after ischaemic stroke and decreases within the first days. Eur J Neurol. 2020; 27:1596-603.

83. Gencer B, Li XS, Gurmu Y, Bonaca MP, Morrow DA, Cohen M, et al. Gut Microbiota-Dependent Trimethylamine N-oxide and Cardiovascular Outcomes in Patients With Prior Myocardial Infarction: A Nested Case Control Study From the PEGASUS-TIMI 54 Trial. J Am Heart Assoc. 2020; 9:e015331.

84. Sun T, Zhang Y, Yin J, Peng X, Zhou L, Huang S, et al. Association of Gut Microbiota-Dependent Metabolite Trimethylamine N-Oxide with First Ischemic Stroke. J Atheroscler Thromb. 2021; 28:320-8.

85. Tang WHW, Li XS, Wu Y, Wang Z, Khaw KT, Wareham NJ, et al. Plasma trimethylamine N-oxide (TMAO) levels predict future risk of coronary artery disease in apparently healthy individuals in the EPIC-Norfolk prospective population study. Am Heart J. 2021; 236:80-6.

86. Bae S, Ulrich CM, Neuhouser ML, Malysheva O, Bailey LB, Xiao L, et al. Plasma choline metabolites and colorectal cancer risk in the Women's Health Initiative Observational Study. Cancer Res. 2014; 74:7442-52.

87. Guertin KA, Li XS, Graubard BI, Albanes D, Weinstein SJ, Goedert JJ, et al. Serum Trimethylamine N-oxide, Carnitine, Choline, and Betaine in Relation to Colorectal Cancer Risk in the Alpha Tocopherol, Beta Carotene Cancer Prevention Study. Cancer Epidemiol Biomarkers Prev. 2017; 26:945-52.

88. Liu X, Liu H, Yuan C, Zhang Y, Wang W, Hu S, et al. Preoperative serum TMAO level is a new prognostic marker for colorectal cancer. Biomark Med. 2017; 11:443-7.

89. Mente A, Chalcraft K, Ak H, Davis AD, Lonn E, Miller R, et al. The Relationship Between Trimethylamine-N-Oxide and Prevalent Cardiovascular Disease in a Multiethnic Population Living in Canada. Can J Cardiol. 2015; 31:1189-94.

90. Rohrmann S, Linseisen J, Allenspach M, von Eckardstein A, Muller D. Plasma Concentrations of Trimethylamine-N-oxide Are Directly Associated with Dairy Food Consumption and Low-Grade Inflammation in a German Adult Population. J Nutr. 2016; 146:283-9.

91. Mafune A, Iwamoto T, Tsutsumi Y, Nakashima A, Yamamoto I, Yokoyama K, et al. Associations among serum trimethylamine-N-oxide (TMAO) levels, kidney function and infarcted coronary artery number in patients undergoing cardiovascular surgery: a cross-sectional study. Clin Exp Nephrol. 2016; 20:731-9.

92. Randrianarisoa E, Lehn-Stefan A, Wang X, Hoene M, Peter A, Heinzmann SS, et al. Relationship of Serum Trimethylamine N-Oxide (TMAO) Levels with early Atherosclerosis in Humans. Sci Rep. 2016; 6:26745.

93. Aslibekyan S, Irvin MR, Hidalgo BA, Perry RT, Jeyarajah EJ, Garcia E, et al. Genome- and CD4+ T-cell methylome-wide association study of circulating trimethylamine-N-oxide in the Genetics of Lipid Lowering Drugs and Diet Network (GOLDN). J Nutr Intermed Metab. 2017; 8:1-7.

94. Kruger R, Merz B, Rist MJ, Ferrario PG, Bub A, Kulling SE, et al. Associations of current diet with plasma and urine TMAO in the KarMeN study: direct and indirect contributions. Mol Nutr Food Res. 2017; 61.

95. Liu X, Xie Z, Sun M, Wang X, Li J, Cui J, et al. Plasma trimethylamine N-oxide is associated with vulnerable plaque characteristics in CAD patients as assessed by optical coherence tomography. Int J Cardiol. 2018; 265:18-23.

96. Matsuzawa Y, Nakahashi H, Konishi M, Sato R, Kawashima C, Kikuchi S, et al. Microbiota-derived Trimethylamine N-oxide Predicts Cardiovascular Risk After STEMI. Sci Rep. 2019; 9:11647.

97. Papandreou C, Bullo M, Zheng Y, Ruiz-Canela M, Yu E, Guasch-Ferre M, et al. Plasma trimethylamine-N-oxide and related metabolites are associated with type 2 diabetes risk in the Prevencion con Dieta Mediterranea (PREDIMED) trial. Am J Clin Nutr. 2018; 108:163-73.

98. Wu C, Xue F, Lian Y, Zhang J, Wu D, Xie N, et al. Relationship between elevated plasma trimethylamine N-oxide levels and increased stroke injury. Neurology. 2020; 94:e667-e77.

99. Roy S, Yuzefpolskaya M, Nandakumar R, Colombo PC, Demmer RT. Plasma Trimethylamine-N-oxide and impaired glucose regulation: Results from The Oral Infections, Glucose Intolerance and Insulin Resistance Study (ORIGINS). PLoS One. 2020; 15:e0227482.

100. Zheng Y, Tang Z, You L, Wu Y, Liu J, Xue J. Trimethylamine-N-oxide is an independent risk factor for hospitalization events in patients receiving maintenance hemodialysis. Ren Fail. 2020; 42:580-6.

101. Mei Z, Chen GC, Wang Z, Usyk M, Yu B, Baeza YV, et al. Dietary factors, gut microbiota, and serum trimethylamine-N-oxide associated with cardiovascular disease in the Hispanic Community Health Study/Study of Latinos. Am J Clin Nutr. 2021; 113:1503-14.

102. Huang X, Li Z, Gao Z, Wang D, Li X, Li Y, et al. Association between risk of preeclampsia and maternal plasma trimethylamine-N-oxide in second trimester and at the time of delivery. BMC Pregnancy Childbirth. 2020; 20:302.

103. Barrea L, Annunziata G, Muscogiuri G, Di Somma C, Laudisio D, Maisto M, et al. Trimethylamine-N-oxide (TMAO) as Novel Potential Biomarker of Early Predictors of Metabolic Syndrome. Nutrients. 2018; 10.

104. Liu W, Wang C, Xia Y, Xia W, Liu G, Ren C, et al. Elevated plasma trimethylamine-N-oxide levels are associated with diabetic retinopathy. Acta Diabetol. 2021; 58:221-9.

105. Liu Y, Guo YL, Meng S, Gao H, Sui LJ, Jin S, et al. Gut microbiota-dependent Trimethylamine N-Oxide are related with hip fracture in postmenopausal women: a matched case-control study. Aging (Albany NY). 2020; 12:10633-41.

106. Chung SJ, Rim JH, Ji D, Lee S, Yoo HS, Jung JH, et al. Gut microbiota-derived metabolite trimethylamine N-oxide as a biomarker in early Parkinson's disease. Nutrition. 2021; 83:111090.

107. Mondul AM, Moore SC, Weinstein SJ, Karoly ED, Sampson JN, Albanes D. Metabolomic analysis of prostate cancer risk in a prospective cohort: The alpha-tocolpherol, beta-carotene cancer prevention (ATBC) study. Int J Cancer. 2015; 137:2124-32.

108. Liu ZY, Tan XY, Li QJ, Liao GC, Fang AP, Zhang DM, et al. Trimethylamine N-oxide, a gut microbiota-dependent metabolite of choline, is positively associated with the risk of primary liver cancer: a case-control study. Nutr Metab (Lond). 2018; 15:81.

109. Huang JY, Luu HN, Butler LM, Midttun O, Ulvik A, Wang R, et al. A prospective evaluation of serum methionine-related metabolites in relation to pancreatic cancer risk in two prospective cohort studies. Int J Cancer. 2020; 147:1917-27.

110. Chen YM, Liu Y, Zhou RF, Chen XL, Wang C, Tan XY, et al. Associations of gut-flora-dependent metabolite trimethylamine-N-oxide, betaine and choline with non-alcoholic fatty liver disease in adults. Sci Rep. 2016; 6:19076.

111. Leon-Mimila P, Villamil-Ramirez H, Li XS, Shih DM, Hui ST, Ocampo-Medina E, et al. Trimethylamine N-oxide levels are associated with NASH in obese subjects with type 2 diabetes. Diabetes Metab. 2021; 47:101183.
